# Nonlinear spatial integration in retinal bipolar cells shapes the encoding of artificial and natural stimuli

**DOI:** 10.1016/j.neuron.2021.03.015

**Published:** 2021-05-19

**Authors:** Helene Marianne Schreyer, Tim Gollisch

**Affiliations:** 1 Department of Ophthalmology, University Medical Center Göttingen, 37073 Göttingen, Germany; 2 Bernstein Center for Computational Neuroscience Göttingen, 37077 Göttingen, Germany

**Keywords:** Retinal bipolar cells, membrane potential recording, nonlinear encoding, spatial integration, linear-nonlinear model, natural stimuli

## Abstract

The retina dissects the visual scene into parallel information channels, which extract specific visual features through nonlinear processing. The first nonlinear stage is typically considered to occur at the output of bipolar cells, resulting from nonlinear transmitter release from synaptic terminals. In contrast, we show here that bipolar cells themselves can act as nonlinear processing elements at the level of their somatic membrane potential. Intracellular recordings from bipolar cells in the salamander retina revealed frequent nonlinear integration of visual signals within bipolar cell receptive field centers, affecting the encoding of artificial and natural stimuli. These nonlinearities provide sensitivity to spatial structure below the scale of bipolar cell receptive fields in both bipolar and downstream ganglion cells and appear to arise at the excitatory input into bipolar cells. Thus, our data suggest that nonlinear signal pooling starts earlier than previously thought: that is, at the input stage of bipolar cells.

## Highlights

• Some retinal bipolar cells represent visual contrast in a nonlinear fashion
• These bipolar cells also nonlinearly integrate visual signals over space
• The spatial nonlinearity affects the encoding of natural stimuli by bipolar cells
• The nonlinearity results from feedforward input, not from feedback inhibition

## Graphical abstract

**Figure:**
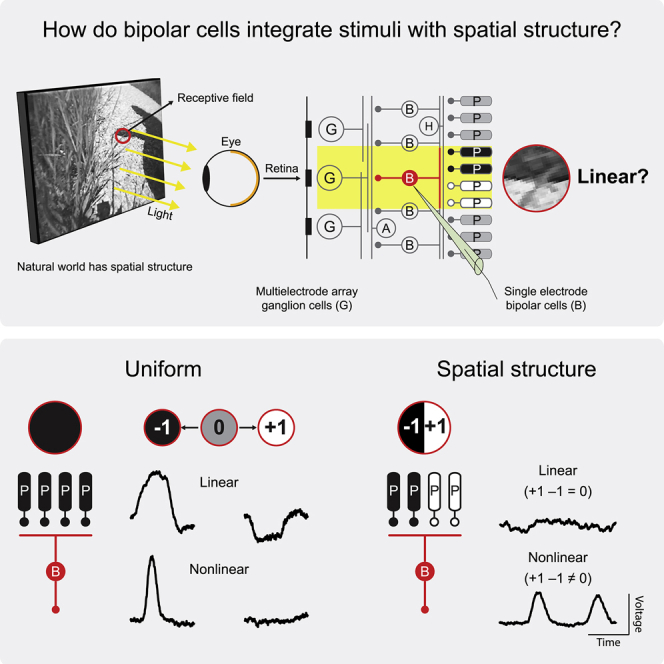

## Teaser

Nonlinear processing in the vertebrate visual system under daylight conditions is often thought to first arise at the output of retinal bipolar cells. Schreyer and Gollisch report that certain bipolar cells in salamander retina already nonlinearly combine their inputs and thereby retain high sensitivity to spatial details of visual stimuli.

## Introduction

Visual processing starts in the eye, where the neural network of the retina parses visual signals into dozens of parallel streams of information, represented by different types of retinal ganglion cells (Baden et al., 2016; Gollisch and Meister, 2010; Masland, 2012; Wässle, 2004). A pivotal role in shaping these streams of information is taken up by retinal bipolar cells, which diversify the photoreceptor signals (Euler et al., 2014; Wässle et al., 2009) and provide the basic excitatory input to ganglion cells (Asari and Meister, 2012; Franke et al., 2017). The nature of bipolar cell signals and their transmission to ganglion cells is crucial for how ganglion cells integrate visual information over their receptive fields. For example, the nonlinear spatial integration that is characteristic for Y-type ganglion cells (Enroth-Cugell and Robson, 1966; Krieger et al., 2017; Petrusca et al., 2007) originates in nonlinear inputs to ganglion cells from bipolar cells (Borghuis et al., 2013; Demb et al., 2001; Schwartz et al., 2012). Moreover, nonlinearities in bipolar-to-ganglion cell signaling lie at the root of different computations and feature extractions performed by specific ganglion cells (Baccus et al., 2008; Gollisch and Meister, 2008, 2010; Krishnamoorthy et al., 2017; Münch et al., 2009; Ölveczky et al., 2003; Zhang et al., 2012) and are crucial ingredients in mechanistic models of contrast adaptation (Jarsky et al., 2011; Ozuysal and Baccus, 2012).

It is typically assumed that these nonlinearities arise in the transmitter release from the axon terminals of bipolar cells in the inner plexiform layer, whereas voltage signals at the soma of bipolar cells should be linearly related to visual contrast (Baccus et al., 2008; Borghuis et al., 2013; Kuo et al., 2016; Shapley, 2009; Turner et al., 2018). This linearity of bipolar cell membrane potential responses is thought to follow from the linear response characteristics of photoreceptors (Baccus and Meister, 2002; Baylor et al., 1974; Rieke, 2001; Tranchina et al., 1991) and the continual, linear release of neurotransmitter by the photoreceptor ribbon synapse in the outer plexiform layer (Heidelberger et al., 2005; Shapley, 2009; Witkovsky et al., 2001). Moreover, current computational models of the retina assume linear receptive fields of bipolar cells, followed by a nonlinear output transformation at their terminals (Baccus et al., 2008; Liu et al., 2017; Schwartz et al., 2012; Turner et al., 2018).

However, there is little data about how bipolar cells represent complex visual signals in their somatic membrane potential and whether the presumed linearity actually holds across the diversity of bipolar cell types. Difficulties for studying bipolar cells arise from their relatively inaccessible location in the retina between the layers of photoreceptors and ganglion cells, their small soma size, and their signaling by graded potentials rather than action potentials. This has rendered bipolar cells an inconvenient target for detailed studies of neural coding. So far, the focus has largely been on studying temporal dynamics of bipolar cells through fairly simple light stimuli, such as uniform spots that were flashed or modulated in time (Awatramani and Slaughter, 2000; Baden et al., 2013a; Euler and Masland, 2000; Franke et al., 2017; Ichinose et al., 2014; Ichinose and Hellmer, 2016; Werblin and Dowling, 1969). An exception has been the mouse rod bipolar cell, which displays a distinct, noise-canceling threshold nonlinearity at the synapse from photoreceptors to the bipolar cell, effective at light intensities near absolute darkness (Berntson et al., 2004; Field and Rieke, 2002; Sampath and Rieke, 2004). This provides sensitivity to individual local inputs. Conceivably, such nonlinear local processing could also be functionally relevant at higher light intensities, yet similar studies for other bipolar cell types have been lacking. Thus, it remains largely unclear if and to what degree bipolar cells, at the level of their membrane potential, contribute to nonlinear signal processing in the retina. Moreover, systematic investigations of bipolar cell responses under more complex visual stimuli, such as spatial patterns or natural stimuli, are still lacking and may provide new insight into bipolar cell stimulus encoding.

In this work, we therefore recorded membrane potentials of bipolar cells in the whole-mount salamander retina under artificial and natural visual stimulation in order to investigate the following questions: (1) Does the bipolar cell membrane potential represent contrast in a nonlinear way? (2) Are there nonlinearities in bipolar cell signal integration? (3) Can bipolar cell responses to artificial and natural stimuli be described by linear filter models? And (4) are nonlinear signals in bipolar cells driven by excitatory or inhibitory inputs?

## Results

We used sharp microelectrodes to intracellularly record somatic voltage signals. To identify bipolar cells, we monitored the recording depth and filled recorded cells with a neuroanatomical tracer for morphological characterization. In addition, retinas were placed onto a multielectrode array to observe ganglion cell spiking activity under current injection into the recorded cell and verify excitatory effects of putative bipolar cells (Figure S1).

### Nonlinear contrast representation in bipolar cells

**Figure 1:**
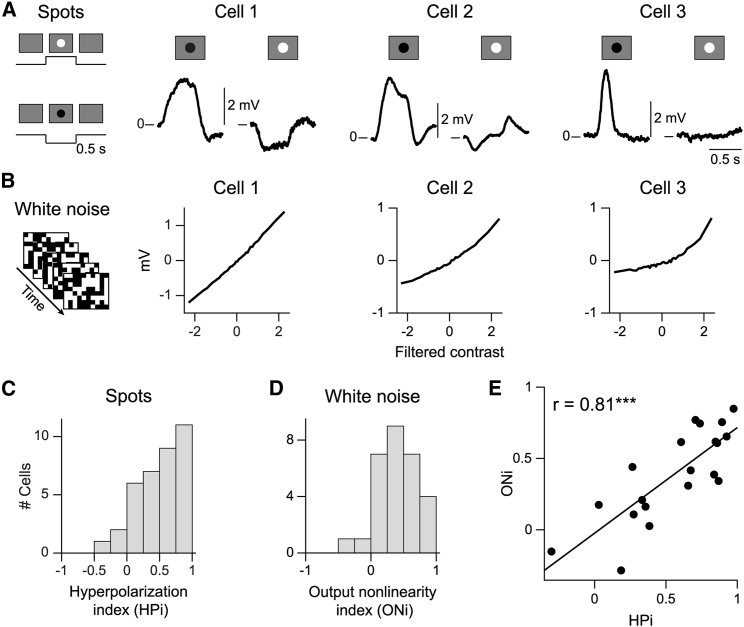
Nonlinear contrast representation in bipolar cells (A) Responses for three sample cells to contrast steps (displayed on the left), shown from spot onset to 500 ms after offset. Here and in subsequent plots, the zero line marks the baseline membrane potential. See Figure S1 for information about recording technique. (B) Output functions under spatiotemporal white noise (as shown on the left) for the same three cells as in (A). (C) Hyperpolarization indices (HPi; n = 36 cells). (D) Output nonlinearity indices (ONi; n = 29 cells). (E) HPi versus ONi (n = 20 cells; black line, linear regression; ^∗∗∗^p < 0.001).

We first tested whether the bipolar cells’ somatic membrane potential represents visual contrast in a linear or nonlinear fashion. We stimulated the cells’ receptive field centers with spots of increased (positive contrast, “white”) or decreased (negative contrast, “black”) light intensity on a gray background. To evaluate elicited membrane potential changes, baseline membrane potentials, as measured during background illumination prior to spot presentation, were subtracted. Figure 1A shows trial-averaged response traces for three sample cells. Cell 1 responded with similar amounts of depolarization and hyperpolarization to preferred and non-preferred spot contrast, respectively, consistent with a linear response. Cell 2 had stronger depolarization than hyperpolarization, suggesting mild nonlinearity. Cell 3 only depolarized and did not show any hyperpolarization for the non-preferred spot, indicative of strongly nonlinear, completely rectifying response characteristics. To quantify the degree of nonlinearly, we computed a hyperpolarization index (HPi) as the normalized difference between the peak depolarization and the peak hyperpolarization. The index is close to zero for linear responses, close to unity for rectifying responses, and negative for larger hyperpolarization than depolarization. Many cells had strongly positive HPi values, providing evidence of nonlinear representation of preferred versus non-preferred contrast (Figure 1C).

We next asked whether similar contrast representation also occurred under continuous, dynamic stimulation. To test this, we applied a spatiotemporal white noise stimulus and analyzed each recorded bipolar cell with a linear-nonlinear (LN) model. The LN model is composed of a linear stimulus filter over space and time and a nonlinear transformation, the cell’s output function, which relates the filtered stimulus signal to the membrane potential. The linear filter represents the cell’s spatiotemporal receptive field and can be obtained from a reverse correlation analysis. The output function is then constructed from the filtered stimulus and the measured membrane potential as a histogram, and its shape directly indicates whether the filtered visual contrast is represented linearly or nonlinearly by the bipolar cell’s membrane potential.

Figure 1B shows the output functions for the same three sample cells as for the contrast steps, ranging again from linear (cell 1) through slightly nonlinear (cell 2) to strongly nonlinear (cell 3). We quantified the degree of nonlinearity in the output function by computing an output nonlinearity index (ONi) that compared the average gain (obtained as the slope of a fitted straight line) in the range of preferred (positive filter output) and non-preferred (negative filter output) contrast sequences. The index is close to zero for linear output functions, larger than zero for rectification, and can be smaller than zero for cells with saturation at large filter output. The distribution of obtained ONi values (Figure 1D) again indicated diverse degrees of nonlinearity, ranging from linear representation to pronounced nonlinear rectification. Furthermore, the degrees of nonlinearity under contrast steps and under white noise were positively correlated (Figure 1E; r = 0.81, p = 2 × 10^−5^, n = 20 cells). Thus, whether a cell represented contrast linearly or nonlinearly did not depend on the applied stimulus but appears to be a cell-specific property.

### Nonlinear spatial integration in bipolar cells

Bipolar cells integrate visual information over space via multiple photoreceptors within their receptive fields. We therefore next asked whether nonlinear operations may also occur at the level of spatial signal integration. To test this, we applied visual stimuli that subdivided the receptive field center into regions of opposing stimulation (“patterned spot”), for example, a bright and a dark half (“split spot”) or four quarters of alternating contrast, and periodically reversed the contrast every half second. If spatial integration occurs linearly, the positive and negative activation from the two opposing contrasts in the patterned spots should cancel each other, and the bipolar cell should not respond to the contrast reversals. Without cancelation, on the other hand, the cell can respond to both reversal directions, resulting in frequency doubling of the response. This frequency doubling is a telltale sign of nonlinear spatial integration and has been frequently used to characterize spatial nonlinearities in ganglion cells (Demb et al., 1999; Enroth-Cugell and Robson, 1966; Hochstein and Shapley, 1976a, 1976b; Petrusca et al., 2007).

**Figure 2:**
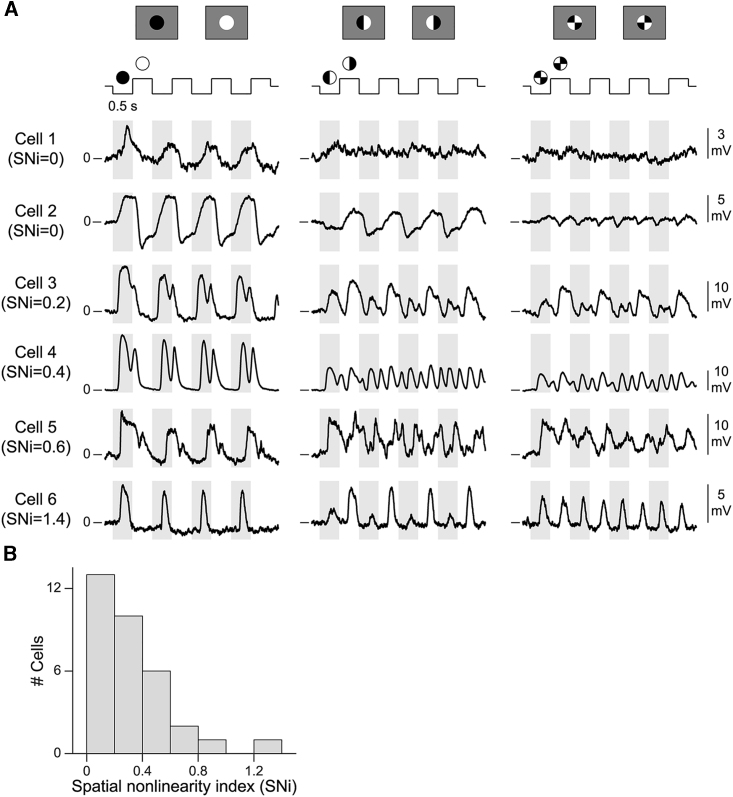
Nonlinear spatial integration in bipolar cells (A) Responses of six bipolar cells to contrast-reversing uniform spots (left), split spots (middle), and patterned spots with four quarters (right column). (B) Spatial nonlinearity indices (SNi; n = 33 cells). See also Figure S2.

Figure 2A shows responses of six bipolar cells to the split spot (middle column) and the spot with four quarters (right column) as well as to a uniform contrast-reversing spot for comparison (left column). All six cells clearly responded to the uniform spot. Yet under the patterned spots, cell 1 showed only small membrane potential fluctuations. Cell 2 also hardly responded to the four-quarters stimulus, and responses to the split spot had no frequency doubling, indicating imperfect stimulus placement with non-symmetric activation by the two stimulus halves. The lack of frequency-doubled responses for these two cells is consistent with linear spatial integration. Cells 3 to 6, on the other hand, responded with depolarization to both reversals, displaying frequency doubling and thus revealing nonlinear spatial integration. The relative sizes of the frequency-doubled responses, compared with responses under the uniform spot, indicate different degrees of nonlinearity, for example modest for cell 3 and pronounced for cell 6.

To quantify the degree of nonlinear stimulus integration, we computed a spatial nonlinearity index (SNi) by comparing the power of the first and higher harmonics in the responses to the uniform and patterned spots (Hochstein and Shapley, 1976b; Turner and Rieke, 2016; see also STAR Methods and Figures S2A and S2B). The index was close to zero if the cell did not respond to the patterned spot reversals, whereas values larger than zero indicated frequency doubling and thus nonlinear stimulus integration. For each cell, we selected the maximum SNi over different spatial patterns. The observed maximum SNi values (Figure 2B) indicate that bipolar cells covered a range from linear (SNi ≈ 0) to clearly nonlinear spatial integration (up to SNi ≈ 1.4).

The stimuli used to test for nonlinear spatial integration were periodic contrast reversals at 1 Hz. It had been shown that light intensity modulations above a few hertz but not at lower temporal frequency can reveal rectified membrane potentials in certain mouse bipolar cells (Ichinose and Hellmer, 2016). We therefore tested whether the observed responses to patterned spots also showed a dependence on temporal frequency. Yet we found that response amplitudes were stable over a wide range of frequencies and decayed at larger frequencies in a similar way as responses to uniform spots (Figures S2C and S2D), indicating that the observed nonlinear spatial integration is independent of the temporal frequency of stimulation.

### Nonlinear spatial integration limits the prediction accuracy of the LN model

To analyze how the observed nonlinearities affect the encoding of visual stimuli, we tested how well bipolar cell responses are captured by the LN model (cf. Figure 1B). The aim of the model is to predict a cell’s response by first performing a linear integration of the stimulus using a spatiotemporal filter and then passing the result through a nonlinear transformation. Thus, the LN model can accommodate nonlinear representations of contrast, but not nonlinear spatial integration.

**Figure 3:**
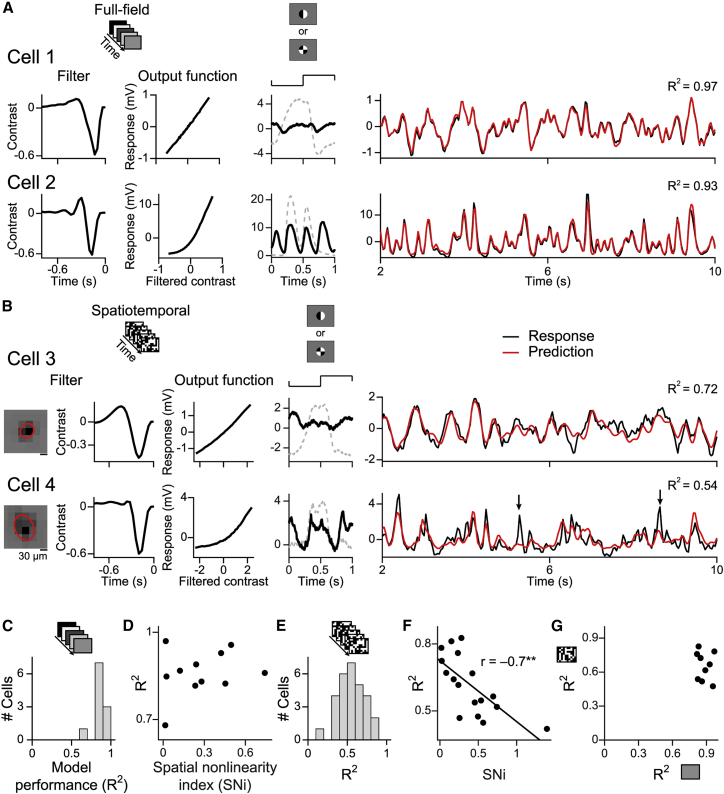
Nonlinear spatial integration limits the prediction accuracy of the linear-nonlinear (LN) model (A) Left: LN model (filters and output functions) for full-field temporal flicker for two sample cells. Middle: responses to the patterned spot with maximal frequency doubling (black) and to the uniform spot (dashed gray) for comparison. Right: measured (black) and predicted (red) responses. (B) Left: LN model (filters and output functions) for spatiotemporal flicker for two sample cells. Middle: same as in (A). Right: measured (black) and predicted (red) responses. Black arrows mark non-predicted responses for cell 4. (C) Model performance (R^2^) under full-field white noise (n = 11 cells). (D) Spatial nonlinearity index versus R^2^ under full-field white noise (n = 11 cells). (E) R^2^ for spatiotemporal white noise (n = 29 cells). (F) Same as (D) for spatiotemporal white noise (black line, linear regression; ^∗∗^p < 0.01). (G) R^2^ under full-field versus spatiotemporal white noise for cells recorded under both stimuli (n = 9 cells).

We first looked at LN models under full-field white noise stimulation without spatial structure. The linear filter and the nonlinearity of the LN model were again obtained with reverse correlation. The stimulus contained a repeatedly inserted fixed white noise sequence (“frozen noise”), which was excluded from the reverse correlation analysis and served as a held-out test stimulus. Figure 3A shows the obtained model components for two sample cells, one spatially linear and the other nonlinear. For both cells, the predicted responses obtained from the LN model accurately matched the cells’ averaged responses to the frozen noise (Figure 3A). To quantify the similarity between prediction and actual response, we computed the explained variance R^2^ as the squared correlation coefficient between model prediction and averaged response. Across the population of recorded cells, this prediction performance ranged from 68% to 97% (Figure 3C), indicating that the LN model accurately predicted responses of bipolar cells to stimuli with no spatial structure.

Next, we investigated whether the accuracy of the prediction was affected by spatial structure, using spatiotemporal white noise. Figure 3B shows the LN model components (spatial and temporal filter as well as nonlinearity) for a spatially linear and a spatially nonlinear sample cell. We used the model to predict voltage traces for several held-out segments. The more linear cell 3 had fairly accurate predictions with 72% explained variance, whereas the more nonlinear cell 4 only yielded 54% explained variance. In general, the distribution of model performance for the spatiotemporal white noise (Figure 3E) was much broader than that for spatially uniform white noise and varied between 13%–83% explained variance (n = 29 cells).

To test whether it was the nonlinear spatial integration that caused low prediction performance of the LN model, we related the SNi to the model performance. For the full-field white noise, we observed no correlation (r = 0.24, p = 0.46, n = 11 cells); spatially linear as well as nonlinear cells showed good model predictions (Figure 3D). For the spatiotemporal white noise, however, we found a clear negative correlation (Figure 3F; r = −0.7, p = 0.002, n = 17 cells); higher spatial nonlinearity indices (i.e., stronger responses to the patterned spots) came with lower performance of the spatiotemporal LN model. Furthermore, we observed that model performances under full-field and spatiotemporal stimulation were not correlated (Figure 3G; r = −0.17, p = 0.66, n = 9 cells) and that, in particular, cells with high model performance under the full-field white noise could have lower model performance under spatiotemporal white noise. This confirms that it is the spatial structure of the stimulus, not the particular sampling of cells, that leads to low response predictions under spatiotemporal stimulation.

The results show that the assumed linear integration of the model does not correctly describe all bipolar cells. For example, the LN model would predict activity cancelation when simultaneous opposing contrast occurs inside the receptive field, which may explain why it missed specific response peaks of nonlinear bipolar cells (see arrows in Figure 3B for cell 4).

### Nonlinear contrast representation and spatial integration under natural movies in bipolar cells

**Figure 4:**
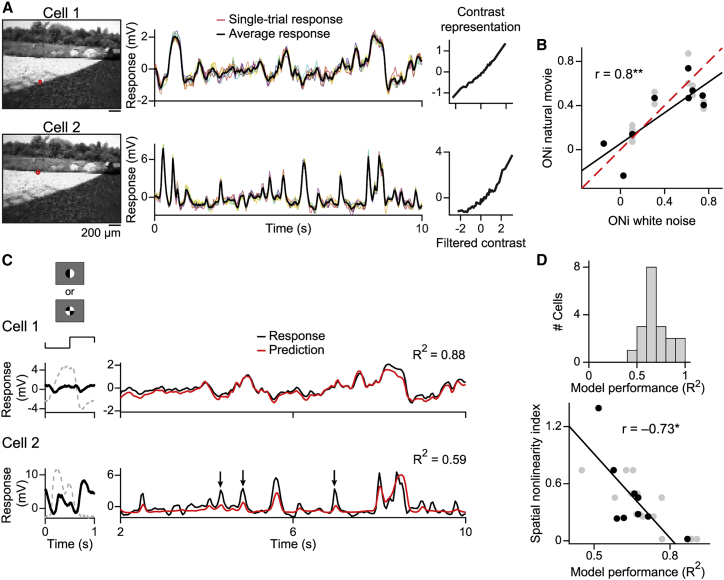
Nonlinear contrast representation and spatial integration under natural movies (A) Responses to natural movies from two sample cells. Left: movie frame with receptive field outlines (red). Middle: single-trial voltage traces (colored lines, 9 or 10 trials) and average response (black lines) to the first 10 s of the movie. Right: output functions obtained from the movie responses. (B) Output nonlinearity index (ONi) under white noise versus under natural movies. Black dots, average ONi over movies for each cell (n = 9 cells); gray dots, ONi for individual movies (n = 19); black line, linear regression (^∗∗^p < 0.01); dashed red line, identity. (C) LN model performance under natural movies for the two sample cells of (A). Left: responses to the patterned spot. Right: responses (black) to the first 10 s of the movie and corresponding prediction (red). R^2^ is the model prediction for the full movie (40 s). Black arrows for cell 2 mark non-predicted responses. (D) Top: model performance (R^2^) over all natural movies (n = 19). Bottom: R^2^ versus spatial nonlinearity index, shown as in B (n = 9 cells, 19 movies, ^∗^p < 0.05).

To test whether bipolar cells represent and integrate contrast also nonlinearly under natural stimuli, we recorded responses to movies from the “CatCam” database (Betsch et al., 2004), which, although they do not represent the natural habitat of salamanders, contain a variety of natural objects, textures, and motion signals. Recorded bipolar cells responded reliably to different trials of such a movie (Figure 4A; time-averaged SD 0.34 ± 0.19 mV, mean ± SD, n = 19 movies, nine cells).

We investigated whether the nonlinear contrast representation observed under spatiotemporal white noise (e.g., cell 3 in Figure 1B) persisted under natural stimulation. To apply the LN model analysis to the natural movies, we kept the spatiotemporal filter from the white noise experiments, assuming that the receptive field remains approximately constant under the different stimulus contexts. (Estimating the filter from the natural-movie responses is not feasible here because of the comparatively small amount of data and the inherent correlation structure of natural stimuli.) We then re-computed the output function from the relation between the filtered movie signal and the measured responses to the movie (Heitman et al., 2016). The obtained output functions showed linear as well as nonlinear representation of contrast under natural movies for different cells (Figure 4A). We again quantified the degree of nonlinearity by computing the ONi as described for white noise stimuli. The ONi under natural stimuli was strongly correlated with the ONi under white noise (Figure 4B; r = 0.8, p = 0.009, n = 9 cells), and the two measures did not differ significantly (p = 0.43, n = 9 cells). Thus, the nonlinear contrast representation of bipolar cells was similar under artificial and natural stimulus statistics.

To study spatial integration with natural movies, we investigated the performance of the LN model for these stimuli. We kept the filter as well as the output function of the model as obtained under spatiotemporal white noise, so as not to use the natural-movie data for both parameter estimation and model evaluation. Figure 4C shows comparisons of model predictions for the two sample cells of Figure 4A. Overall, model performance for natural movies (Figure 4D, top) was somewhat higher than for spatiotemporal white noise, but still with considerable spread, ranging between 45% and 91% explained variance (n = 19 movies, nine cells). To test whether, as for the spatiotemporal white noise, it was the nonlinear spatial integration that caused low prediction performance for some cells, we again related the SNi to the model performance. We observed a clear negative correlation (Figure 4D, bottom; r = −0.73, p = 0.027, n = 9 cells). Thus, nonlinear spatial integration also limited the predictive quality of the LN model for some bipolar cells under natural stimuli.

### Nonlinear spatial integration in bipolar cells is driven by excitatory feedforward inputs

What may be the biological source of the nonlinear spatial integration? From photoreceptors, bipolar cells receive excitatory feedforward inputs through cation channels (Maple and Wu, 1996), which are integrated along the dendrites and in the soma of the bipolar cells. However, bipolar cells also receive inhibitory feedback from amacrine cells at the bipolar cell synaptic terminals. As bipolar cells are electrically quite compact, these inhibitory signals might back-propagate to the soma and influence the somatically recorded membrane potential (Euler and Masland, 2000; Masland, 2012). Thus, nonlinear signal integration in bipolar cells could result from inhibitory amacrine cell inputs, in particular as amacrine cells might themselves be spatially nonlinear through rectified bipolar cell input, in the same way as traditionally considered for ganglion cells.

**Figure 5:**
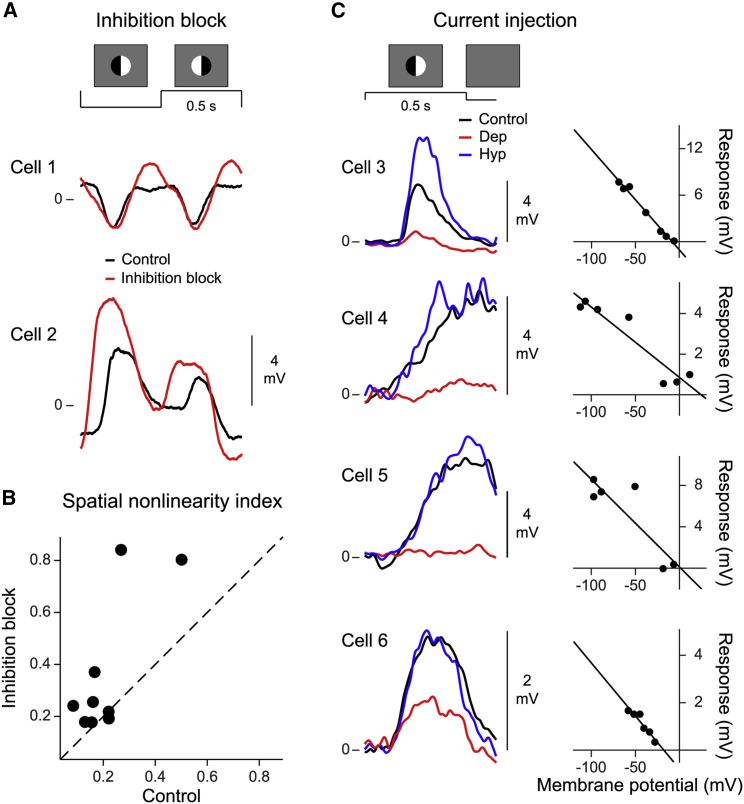
Spatial integration in bipolar cells is driven by excitatory inputs (A) Split-spot responses for two sample cells before (black) and during inhibition block (red). (B) Spatial nonlinearity indices before and during inhibition block (n = 9 cells; dashed line, identity). (C) Left: split-spot responses for four sample cells while injecting depolarizing (red), hyperpolarizing (blue), or no current (black). Right: maximum response amplitude versus baseline membrane potential for each cell (black lines, linear regression; average extrapolated reversal potential: 0 ± 18 mV, mean ± SD).

However, when we tested for a contribution of inhibitory inputs to nonlinear spatial integration in bipolar cells by applying blockers of inhibition, we saw no effect. Frequency doubling in bipolar cell responses to contrast-reversing patterned spots persisted under a cocktail of picrotoxin and strychnine, which blocks GABA_A_, GABA_C_, and glycine receptors (Figure 5A). In fact, spatial nonlinearity indices were rather increased in the presence of the inhibition block (Figure 5B, bottom), similar to observations by Demb et al. (2001) in ganglion cells. The persistence of frequency doubling under inhibition block indicates that inhibitory inputs are not essential for the occurrence of nonlinear spatial integration in bipolar cells.

To further examine the nature of the inputs that underlie nonlinear spatial integration, we aimed to assess the effects of depolarizing or hyperpolarizing the bipolar cell and to estimate the reversal potential of the relevant inputs. Given the difficulty of obtaining whole-cell patch-clamp recordings from the small bipolar cells in a retinal whole-mount preparation in which the cells are covered by other neural layers, we opted for depolarization and hyperpolarization in current clamp by injecting positive and negative currents through the sharp microelectrode. Analyzing how the measured stimulus-evoked response amplitude depends on the baseline membrane potential under current injection can reveal whether the responses are driven by excitatory or inhibitory inputs (Zaghloul et al., 2003). If excitatory cation inputs caused the responses to the patterned spot, positive (depolarizing) current injection should decrease the driving force of the input and thus reduce the measured voltage response, whereas inhibition-driven responses should increase with positive current injection. We observed that the former was the case; for all four recorded bipolar cells, responses to flashed split spots decreased with more depolarizing current injection, and extrapolation suggested a sign reversal of the responses near 0 mV baseline membrane potential (Figure 5C). Thus, the responses that are characteristic for nonlinear spatial integration are driven by excitatory, cationic inputs, corroborating that nonlinear spatial integration does not depend on inhibitory signals.

### Input nonlinearities dominate the nonlinear characteristics of bipolar cell responses

**Figure 6:**
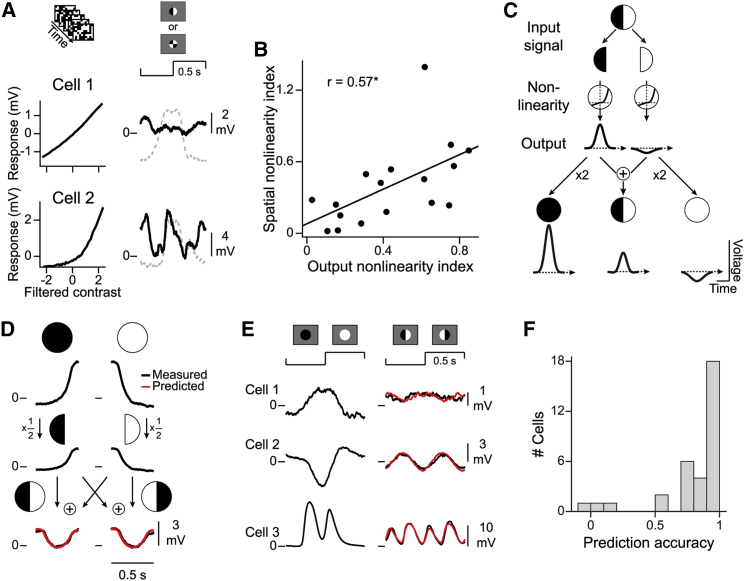
Input nonlinearities dominate the nonlinear characteristics of bipolar cells (A) Output functions and responses to patterned spots for two sample cells. (B) Output nonlinearity indices (absolute values) versus spatial nonlinearity indices (n = 17 cells; black line, linear regression; ^∗^p < 0.05). (C) Relationship between responses to the split spot and the uniform spot under a model with only local nonlinearities. The local input signals are passed through a nonlinearity. For the split spot (middle), the two outputs are summed; for a uniform spot, the corresponding output is doubled (left and right). (D) Predicting the split-spot response for a sample cell. Top: measured response to contrast-reversing uniform spots, split into two half-periods. Middle: estimated response to half-field black and white spots (half of uniform-spot responses). Bottom: predicted (red) and measured response (black) to the split spot, obtained by summing the half-field responses. (E) Responses to uniform and split spots, together with the split-spot predictions (red) for three further sample cells. See Figure S3 for additional examples. (F) Prediction accuracy of the model (n = 33 cells).

Given that bipolar cells showed both nonlinear contrast representation (Figure 1) as well as nonlinear spatial integration (Figure 2), we asked whether these two nonlinearities are related. For example, the first sample cell in Figure 6A had a near linear contrast representation as well as approximately linear spatial integration. The second sample cell, on the other hand, displayed strongly nonlinear characteristics in both cases. Across all cells, the ONi and the SNi were indeed positively correlated (r = 0.57, p = 0.017, n = 17 cells; Figure 6B).

The correspondence between the two measures of nonlinearity let us hypothesize that they have the same origin, namely, a local nonlinear transformation of excitatory inputs. The nonlinear contrast representation would then simply be inherited from the local transformation with no second nonlinear stage acting on the integrated signal. If this is the case, the responses to the split spot and to the uniform spot should be related through a simple, generic model (Figure 6C). In the model, the contrast signal from each half of the receptive field is filtered and transformed by a local nonlinear function, and the two resulting signals are linearly summed to yield the membrane potential without any further nonlinearity. Therefore, according to the model, the split spot response is the sum of the responses to a half-field white stimulus and a half-field black stimulus, which, in turn, are half of the responses to the full white and full black spots, respectively. This lets us predict the responses to the contrast-reversing split spot without having to explicitly take nonlinear transformations into account because the measured responses to the uniform spots already contain the local nonlinearities that also affect the stimulus integration for the split spot. Note that the same prediction is obtained for the patterned spot with four quarters, as it effectively also comprises two receptive field halves of increasing and decreasing light intensity, respectively.

Figure 6D illustrates the procedure for the prediction of a sample cell that displayed nonlinear spatial integration. Indeed, we found that the predicted trace (red line) matched the measured response trace (black line) quite accurately. Figure 6E shows the responses and predicted traces of three further cells. For the first, the predicted response was small, similar to the cell’s actual response. The other two cells had larger response predictions (indicative of nonlinear spatial integration), which matched the measured responses (see Figure S3 for more sample cells.) To quantify whether this simple model captured the responses under patterned-spot stimulation, we computed a prediction accuracy measure, which takes a value of unity if the predicted and measured responses match and zero for a mismatch. For most of our cells (28 of 33), we observed high prediction accuracy, with values larger than 0.7 (Figure 6F). For the three cells with poor prediction accuracy (<0.2 in Figure 6F), the failure appears to have resulted from unbalanced responses to the two reversal direction (whereas the model assumes that both reversals have identical effects), owing to a misalignment of stimulus and receptive field (Figure S3B). Altogether, this analysis suggests that the primary nonlinear signal transformations indeed occur locally before spatial integration at the bipolar cell soma.

### Spatial scale of nonlinear signal processing

**Figure 7:**
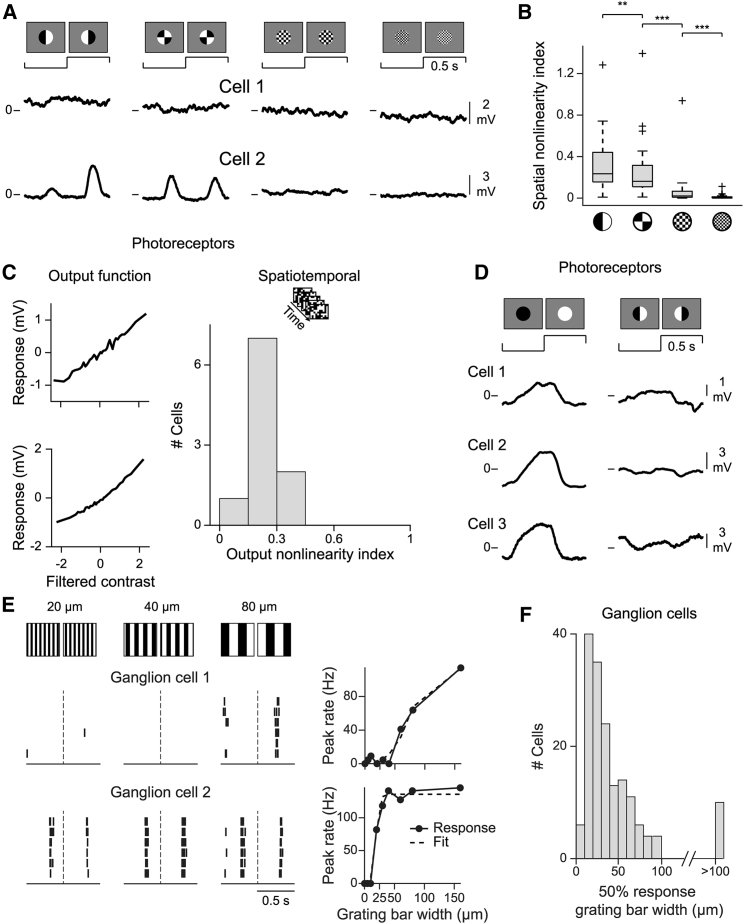
Spatial scale of nonlinearities in bipolar and ganglion cells (A) Responses of two sample bipolar cells to different patterned spots. (B) Spatial nonlinearity indices for the different spatial structures (n = 22 cells for 10 μm squares, n = 33 cells for all others). Box marks the 25th and 75th percentiles, the central line the median, the whiskers the range of data, and crosses the outliers (according to Tukey’s fences). ^∗∗^p < 0.01 and ^∗∗∗^p < 0.001. (C) Sample photoreceptor output functions under spatiotemporal white noise and output nonlinearity indices for photoreceptors (n = 10 cells). (D) Photoreceptor responses to contrast-reversing uniform and split spots. (E) Left: spike rasters of two sample ganglion cells under 1 Hz reversing gratings with different bar widths (indicated on top). Each row is a temporal period (dashed line, reversal in the middle of the period). The second cell already responded for a bar width of 20 μm. Right: peak firing rate versus grating width, fitted by a logistic function, whose midpoint defined the scale of spatial sensitivity. (F) Extracted spatial scales (n = 167 ganglion cells).

The nonlinear spatial integration suggests that bipolar cells are sensitive to spatial structure below the scale of their receptive fields. To explore this spatial resolution, we investigated the spatial scale at which nonlinear integration appears. We stimulated receptive field centers with contrast-reversing patterned spots of increasingly finer spatial structure, using spots divided into two halved, into four quarters, and into squares of 25 or 10 μm in length (Figure 7A). Responses to fine checkerboards of 25 μm were substantially reduced, and 10 μm checkerboards yielded essentially no responses (see Figure 7B), indicating that such fine spatial patterns are integrated linearly. In contrast, for subdivisions of the receptive field into quarters, responses were still nearly as strong as for the split spot, indicating that nonlinear spatial integration can occur at a scale of about 30 μm.

Although photoreceptors in the salamander are somewhat smaller than this scale, with typical sizes of 10–15 μm (Mariani, 1986; Sherry et al., 1998), their spacing may be larger, making them a potential source of the nonlinearity. Indeed, nonlinear responses have previously been observed in cone photoreceptors, at least when high enough visual contrast was provided (Endeman and Kamermans, 2010; Howlett et al., 2017). To test whether photoreceptors display nonlinearities for stimuli used in the present study, we intracellularly measured their voltage responses under spatiotemporal white noise stimulation and reversing uniform and split spots. We found that photoreceptors, compared with bipolar cells (cf. Figures 1D and 2B), showed more linear and less diverse output functions (Figure 7C) and also integrated signals linearly (Figure 7D). Thus, photoreceptor membrane potentials seem not be the origin of the nonlinearity in the input to bipolar cells under the applied stimulus conditions.

Given that we found bipolar cells to be sensitive to spatial structure smaller than their receptive fields (typically ∼50–120 μm in salamander; see Figure S4E and Baccus et al., 2008, and Ölveczky et al., 2003), we asked whether this spatial sensitivity translated to ganglion cells. We recorded ganglion cell responses to contrast-reversing square-wave gratings of different spatial frequencies and indeed observed that many ganglion cells responded already to gratings with a bar width as small as 20 μm (Figures 7E and 7F). This high spatial resolution is inconsistent with models in which the first nonlinearity occurs only at the synaptic output of bipolar cells. Moreover, it indicates that the effects of nonlinear spatial integration observed in bipolar cells are preserved along the synaptic transmission to ganglion cells, thereby setting the spatial sensitivity of the retina’s output and mediating retinal responses to stimulus structures of few tens of micrometers.

### Relation of nonlinear processing and standard response properties in bipolar cells

The type of glutamate receptor at the bipolar cell dendrite determines the cell’s preferred contrast (ON versus OFF) and can also influence whether its light responses have transient or sustained characteristics (DeVries, 2000; DeVries and Schwartz, 1999; Euler et al., 2014; Wässle, 2004). It has also been hypothesized that the glutamate receptor could contribute to nonlinear transformations (Demb et al., 2001). We thus aimed to relate a cell’s nonlinearity to its standard response properties.

**Figure 8:**
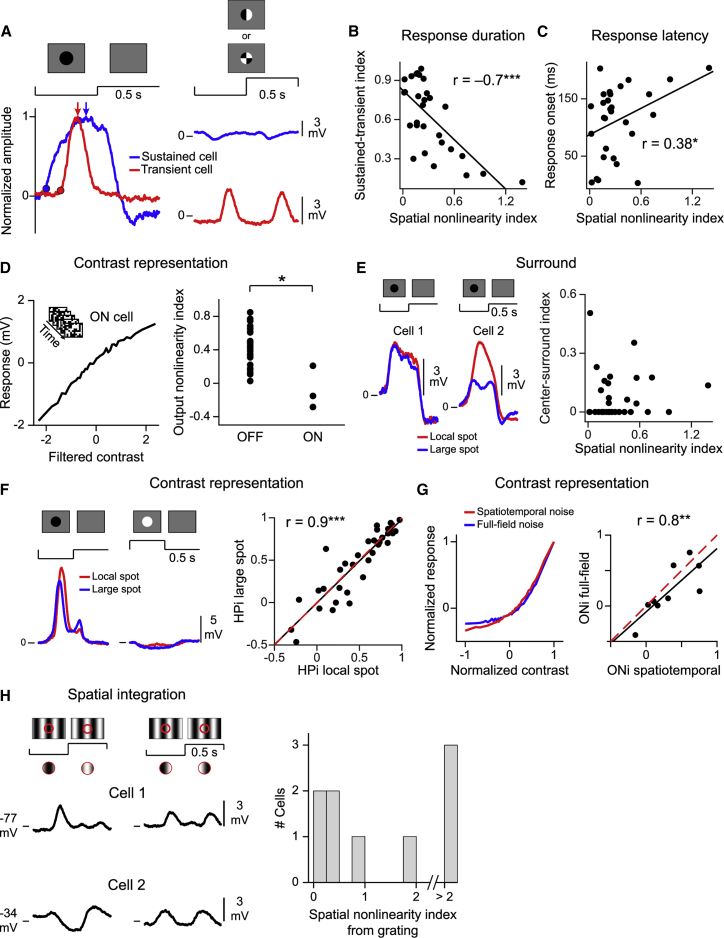
Relation of nonlinearities and standard response properties in bipolar cells (A) Left: responses of two bipolar cells to uniform spots (dots, response onsets; arrows, response peaks). Right: responses for the same cells to the patterned spot with maximal frequency doubling. (B) Spatial nonlinearity indices versus sustained-transient indices (n = 29 cells; black line, linear regression). (C) Same as (B) for response onset. (D) Saturating output function under white noise of an ON bipolar cell (left) and output nonlinearity indices for OFF and ON cells (right; 26 OFF and 3 ON cells). (E) Left: responses to local (red) and large spots (blue) for two sample cells. Right: same as (B) for the center-surround index (n = 33 cells). (F) Left: responses to local (red) and large spots (blue) under preferred and non-preferred contrast for a sample cell. Right: hyperpolarization indices (HPi) for local versus large spots (n = 36 cells; red dashed line, identity). (G) Left: output functions (normalized to maximum and a range of −1 to 1) under spatiotemporal (red) and full-field (blue) white noise for a sample cell. Right: output nonlinearity index (ONi) for spatiotemporal versus full-field white noise (n = 9 cells). (H) Left: response of two cells to different phases of full-field reversing gratings (red circles, schematized receptive field stimulation). Right: distribution of the spatial nonlinearity index under grating stimulation (n = 9 cells). See Figure S4 for further comparisons. ^∗^p < 0.05, ^∗∗^p < 0.01, and ^∗∗∗^p < 0.001.

To assess transient versus sustained responses, we quantified the response kinetics under spots of preferred contrast (Figure 8A) with a sustained-transient index (STi), which takes values close to zero for transient and near unity for sustained responses. The STi was negatively correlated with the SNi (Figure 8B; r = −0.7, p = 3 × 10^−5^, n = 29 cells), showing that more transient cells had stronger nonlinearities as revealed by the patterned-spot experiments. We also checked whether nonlinear response characteristics were related to response latency. Latency can be measured as response onset or time to peak (Franke et al., 2017; Krieger et al., 2017), and we observed that sustained cells showed a fast response onset but longer time to peak, whereas transient cells showed a more sluggish onset but earlier peak (see sample cells in Figure 8A and Figure S4B). Indeed, the two latency measures showed different correlations with the degree of spatial nonlinearity. Response onset was positively correlated with the SNi (Figure 8C; r = 0.38, p = 0.04, n = 29 cells), whereas time to peak indicated a rather negative yet non-significant correlation (r = −0.28, p = 0.14, n = 29 cells). Thus, nonlinear bipolar cells are typically transient cells with a slow onset but perhaps faster peak response.

In addition, we checked whether there were systematic differences in nonlinear properties between ON and OFF bipolar cells. Note, however, that the salamander retina is biased toward OFF cells (Burkhardt et al., 1998; Hare et al., 1986; Hare and Owen, 1990; Segev et al., 2006), and our recordings yielded around 90% OFF and only 10% ON bipolar cells. The few ON cells showed comparatively smaller output nonlinearity indices (p = 0.016, n = 29 cells; Figure 8D) and indicated a saturation of the output function, leading to negative ONi values.

We further examined the suppressive surround of bipolar cell receptive fields and quantified the surround strength by a center-surround index, computed from the peak responses to large and small spots (Figure 8E). Index values larger than zero indicated surround suppression. The center-surround index, however, was not correlated to the SNi (r = 0.05, p = 0.78, n = 33 cells; Figure 8E); cells both with and without suppressive surround could be nonlinear. Consistent with this finding, the nonlinearities of bipolar cells did not depend on the stimulation of the receptive field surround. The relative amplitudes of hyperpolarization and depolarization for black and white spots were similar for small and large spots (r = 0.9, p = 3.6 × 10^−14^, n = 36 cells; Figure 8F), and nonlinearities in the LN model were nearly identical under spatiotemporal and spatially homogeneous white noise, though the latter provides stronger surround activation (Figure 8G; correlation between ONi values: r = 0.8, p = 0.01, n = 9 cells). We also tested full-field stimulation with classical contrast-reversing sinusoidal gratings (Enroth-Cugell and Robson, 1966) and found that they yielded similar results (in particular frequency doubling) as the local stimulation with reversing patterned spots (see Figure 8H and Figures S4C–S4H for further comparisons).

## Discussion

### Nonlinear signal integration in bipolar cells

Our finding of nonlinear spatial integration in bipolar cells clashes with the classical view of linear signal processing in these cells. In particular, bipolar cells are considered to pool their inputs over space in a linear fashion. This view is based on the linear photoreceptor responses and the continuous transmitter release of the ribbon synapses at the photoreceptor terminals (Shapley, 2009). The first nonlinear signal transformation is then thought to occur at the output of bipolar cells, through nonlinear glutamate release from the synaptic terminals (Baccus et al., 2008; Borghuis et al., 2013; Roska and Meister, 2014). The nonlinear integration observed in our work is fundamentally different from this nonlinearity at bipolar cell terminals. From a functional perspective, the nonlinear signal integration—unlike nonlinear processing occurring at the output, after signal integration—allows the retina to maintain sensitivity to spatial frequencies below the scale of bipolar cell receptive fields.

Few studies had previously tested the assumed linear signal pooling in bipolar cells. Accurate predictions of responses to jittering gratings with a linear-receptive-field model for sample bipolar cells (Baccus et al., 2008) supported linear integration, yet leaving room for differences in integration characteristics across bipolar cell types. Support for linear spatial integration in the mouse retina came from measurements of bipolar cell glutamate release, which revealed response nulling under reversing gratings (Borghuis et al., 2013). Yet, as this study focused on bipolar cells connected to alpha-type ganglion cells, whose major inputs likely come from few specific bipolar cell types (Schwartz et al., 2012; Tien et al., 2017; Yu et al., 2018), other bipolar cells in the mouse retina may still differ in this respect.

It is thus worth noting that there is some evidence for nonlinear signal integration in mammalian bipolar cells as well. Rod bipolar cells in the mouse retina integrate inputs nonlinearly near absolute darkness (Berntson et al., 2004; Field and Rieke, 2002; Sampath and Rieke, 2004), though this is typically considered a rather specific scenario in which the nonlinearity depends on the saturated state of the photoreceptor-to-bipolar cell signal transmission in complete darkness. Under photopic conditions, Freeman et al. (2015) observed that responses of primate ganglion cells did not completely cancel out when two cones projecting onto the same bipolar cell were stimulated with opposing contrast. The authors attributed the remaining response to nonlinearities in cone photoreceptors, leading to nonlinear integration by the bipolar cell, in line with our findings. Furthermore, some ganglion cells in mouse retina are sensitive to patterns with spatial scales of about 20 μm (Jacoby and Schwartz, 2017; Krieger et al., 2017; Mani and Schwartz, 2017; Schwartz et al., 2012; Zhang et al., 2012), though this high spatial sensitivity was interpreted as resulting from spatially linear bipolar cells with small receptive fields and nonlinear synaptic release. Yet given that reports of receptive field sizes of mouse bipolar cells are typically in the range of 40–80 μm (Berntson and Taylor, 2000; Borghuis et al., 2013; Franke et al., 2017; Schwartz et al., 2012), spatially nonlinear bipolar cells could provide a viable alternative source of the observed spatial sensitivity.

Regarding representation of visual contrast by the bipolar cell membrane potential, several previous studies had shown linear characteristics, mainly in the context of contrast adaptation (Baccus and Meister, 2002; Rieke, 2001; Sakai and Naka, 1987a, 1987b; Toyoda, 1974), yet often relying on few sample cells. Other studies in the salamander and mammalian retina had documented somatic nonlinearities in the membrane potential (Burkhardt et al., 2011; Burkhardt and Fahey, 1998; Euler and Masland, 2000; Fahey and Burkhardt, 2003; Ichinose and Hellmer, 2016; Molnar et al., 2009; Wu et al., 2000), but often based on a much larger applied luminance or contrast range compared with the present study. Under large light intensity changes, nonlinear output functions are expected because of saturation effects. In the present work, nonlinearities were measured with moderate light intensity changes (e.g., as occurring within a single natural scene), indicating that nonlinearities are relevant not only when changing luminance regimes but also for stimulus encoding within a natural range of Weber contrast values.

Taken together, our results support a bipolar cell model in which the input signals can be nonlinearly transformed, leading to nonlinear spatial integration and nonlinear contrast representation at the soma of some bipolar cells and in which additional rectification of the integrated signal occurs at the axon terminals (Demb et al., 2001). Regarding the representation of contrast, both these nonlinear mechanisms typically provide rectifying transformations, and the combined, enhanced nonlinearity might be required for optimal encoding of natural contrast ranges, as previously hypothesized (Burkhardt et al., 2006). An exception may be seen in the few recorded ON bipolar cells of our study, two of which displayed stronger hyperpolarization than depolarization (Figure 8D), which might effectively provide partial cancelation of rectification occurring in the synaptic release.

### Putative mechanisms for nonlinearities in bipolar cells

The observed nonlinearities appear to arise along the feedforward inputs before signal integration in the bipolar cell soma (Figures 5 and 6). A dependence on inhibitory inputs through back-propagating amacrine cell inputs (Euler and Masland, 2000; Masland, 2012) or through glycinergic interplexiform cells (Jiang et al., 2014; Maple and Wu, 1998) is unlikely, as inhibition block had little effect on the nonlinear spatial integration and the relevant responses displayed a reversal potential near 0 mV, indicative of a cation-mediated input. In addition, the nonlinear signal integration in bipolar cells led to depolarizing rather than hyperpolarizing events under patterned spots (e.g., Figure 2A), which is inconsistent with a simple inhibitory input and would require a disinhibitory interaction. Finally, the observed spatial sensitivity of retinal ganglion cells to gratings with bar widths as small as 20 μm suggests that the bipolar cell layer, prior to interactions with amacrine and ganglion cells, must provide spatial sensitivity below the scale of bipolar cell receptive fields (about 50 μm and larger; Figure S4E; Baccus et al., 2008; Ölveczky et al., 2003). As amacrine cells receive their signals through the bipolar cells, they could not create sensitivity to spatial structures below the bipolar cell receptive field scale if all signals below this scale were filtered out by the bipolar cells.

The signal transformations underlying the nonlinear spatial integration thus appear to originate in the outer retina layers. One potential site could be the bipolar cell dendrites (Demb et al., 2001), where different glutamate receptors diversify the photoreceptor signals into ON and OFF (metabotropic versus ionotropic receptors) as well as transient and sustained (different types of ionotropic receptors) signals (Borghuis et al., 2014; DeVries, 2000; DeVries and Schwartz, 1999; Maple et al., 1999). Along this line, we found that transient bipolar cells were more nonlinear compared with sustained cells (Figure 8B), and only ON bipolar cells showed response saturation (Figure 8D). Thus, differences in receptor dynamics or in dendritic ion channels, for example, voltage-dependent sodium channels (Zenisek et al., 2001), could lead to the different degrees of nonlinearities.

Alternatively, presynaptic mechanisms in the photoreceptors’ synaptic terminals might provide a potential source of nonlinearities. Nonlinear calcium signals have indeed been observed at some mouse cone terminals (Baden et al., 2013b). Such a nonlinearity at photoreceptor synapses would still be consistent with linear somatic voltage signals that we measured in photoreceptors (Figures 7C and 7D). Depending on light level and contrast range, nonlinear voltage signals and adaptation in photoreceptors (Clark et al., 2013; Endeman and Kamermans, 2010; Howlett et al., 2017) could also contribute to nonlinear spatial integration in bipolar cells, though our photoreceptor recordings did not provide evidence for this under the stimulus conditions used here. Finally, nonlinear integration in bipolar cells could arise from temporal differences between increases and decreases in the photoreceptors’ voltage or glutamate responses, as the temporal offset would prevent exact cancelation of signals from activated and deactivated photoreceptors (see Borghuis et al., 2013, for a similar idea for bipolar-to-ganglion cell signaling). Note, though, that such presynaptic mechanisms would require that different photoreceptor types process signals differently (some linearly, some nonlinearly) and that different bipolar cells pool signals preferentially from different photoreceptor types, so that the observed diversity of nonlinear properties among bipolar cells could be explained.

In addition, species-specific features might influence the observed nonlinearities. For example, the salamander retina has more photoreceptor types (Sherry et al., 1998), and bipolar cell morphology might be more diverse (∼12–20 types; Pang et al., 2004; Wu et al., 2000) compared with the mouse retina (Euler et al., 2014). To further investigate the relevant mechanisms, a better identification of subtypes of salamander bipolar cells would be helpful. Unfortunately, despite existing morphological and physiological characterizations of bipolar cells in the salamander retina (Hare et al., 1986; Wu et al., 2000), clear identification procedures of subtypes or unambiguous anatomical or genetic characterizations are still lacking. Purely functional characterizations of bipolar cells, as also applied in the present work, can be used to make headway (Asari and Meister, 2012, 2014), though more detailed characterizations and morphological information will likely be needed to clearly separate types (Franke et al., 2017; Wu et al., 2000).

### Functional relevance of nonlinear computations in bipolar cells

The retina extracts diverse features from the visual scene, which are represented by the activity of different types of ganglion cells at the retina’s output. This feature extraction depends on subunits within the ganglion cell receptive field, which correspond to the presynaptic bipolar cells and which are thought to provide local contrast information for nonlinear computations (Baccus et al., 2008; Demb et al., 2001; Freeman et al., 2015; Gollisch, 2013; Gollisch and Meister, 2010; Hochstein and Shapley, 1976a; Liu et al., 2017; Schwartz and Rieke, 2011; Schwartz et al., 2012). The nonlinear spatial integration we observed in bipolar cells could extend this view of subunits (e.g., through local nonlinearities inside subunits) and provide ganglion cells with feature sensitivity on a spatial scale below the size of bipolar cell receptive fields (Figures 7E and 7F). In dim light conditions near complete darkness, for example, retinal ganglion cells as well as human observers can report the detection of single photons by rod photoreceptors (Barlow et al., 1971; Hecht et al., 1941). The high sensitivity is thought to come through nonlinear signal integration in rod bipolar cells (Berntson et al., 2004; Field and Rieke, 2002; Sampath and Rieke, 2004). Our findings could point to a similar functional mechanism during daylight where local changes that happen below the scale of bipolar cell receptive fields are reported to ganglion cells and the brain. This maintains sensitivity to small spatial structures despite the signal convergence from photoreceptors to ganglion cells and could be particularly relevant for animals whose retinal cells have large receptive fields in terms of visual angle (e.g., because their eyes are small).

## STAR★Methods

### Key resources table

**Table:** 

REAGENT or RESOURCE	SOURCE	IDENTIFIER
**Chemicals, peptides, and recombinant proteins**		

Neurobiotin	Vector Laboratories	Cat# SP-1120; RRID: AB_2313575
Streptavidin, Alexa Fluor 488 conjugate	Thermo Fisher Scientific	Cat# S11223
To-Pro-3 Iodide (642/661)	Thermo Fisher Scientific	Cat# T3605
Formaldehyde	Sigma-Aldrich	Cat# F8775
Triton X-100	Sigma-Aldrich	Cat# T8787
Picrotoxin	Sigma-Aldrich	Cat# P1675
Strychnine	Sigma-Aldrich	Cat# S0532
CNQX	Biotrend AG	Cat# BN0154
D(+)-Glucose monohydrate	Carl Roth	Cat# 6887.1
NaCl	Merck	Cat# S7653
KCl	Merck	Cat# 1049360250
MgCl_2_	Merck	Cat# M2670
CaCl_2_	Sigma-Aldrich	Cat# 223506
NaHCO_3_	Merck	Cat# 1063290500

**Deposited data**		

Recorded voltage traces of bipolar cells under visual stimulation in vitro	This paper	https://gin.g-node.org/gollischlab/Schreyer_and_Gollisch_2021_salamander_retinal_bipolar_cell_recordings

**Experimental models: Organisms/strains**		

Ambystoma mexicanum, adult pigmented wild-type, both sexes	Axolotl Facility, Center for Regenerative Therapies Dresden, Germany, and Adem Morankic, Reutlingen, Gemany	N/A

**Software and algorithms**		

IGOR Pro	WaveMetrics	RRID: SCR_000325
MATLAB	Mathworks	RRID: SCR_001622
Fiji	Schindelin et al. (2012)	https://fiji.sc; RRID: SCR_002285
Custom spike sorting algorithm in IGOR Pro	adapted from Pouzat et al. (2002)	N/A
pCLAMP 10	Molecular Devices	https://www.moleculardevices.com/products/software/pclamp.html; RRID:SCR_011323
MC Rack	Multi Channel Systems	https://www.multichannelsystems.com/software/mc-rack; RRID:SCR_014955

**Other**		

Natural movies from “CatCam” database	Betsch et al. (2004)	https://zenodo.org/record/46481

### Resource availability

#### Lead contact

Further information and requests for resources and reagents should be directed to and will be fulfilled by the Lead Contact, Tim Gollisch (tim.gollisch@med.uni-goettingen.de).

#### Materials availability

This study did not generate new unique reagents.

#### Data and code availability

The recorded voltage traces of bipolar cells and information about the corresponding visual stimuli as well as sample code for analysis are available at

https://gin.g-node.org/gollischlab/Schreyer_and_Gollisch_2021_salamander_retinal_bipolar_cell_recordings.

### Experimental model and subject details

#### Animals

All experimental procedures were in accordance with national and institutional guidelines and were approved by the University Medical Center Göttingen (protocol number T11/35). In total, 28 adult axolotl salamanders (*Ambystoma mexicanum*; pigmented wild-type, both sexes) of minimum 12 months of age (exact birthdates not reported by supplier) were used. Axolotls were held in standard aquarium basins with burrow-like hide-out places (12-hour light-dark cycle; 1-2 animals per basin), fed daily, and constantly supplied with filtered water.

### Method details

#### Electrophysiology

The eyes of dark-adapted (∼1 h) axolotl salamanders were enucleated, and the vitreous humor was carefully removed. In 19 experiments, the whole retina was detached from the pigmented epithelium, mounted over a hole of 1.5-2 mm diameter on a nitrocellulose filter membrane and placed ganglion cell-side-down on a 60-channel perforated multielectrode array (MEA) (Reinhard et al., 2014). A pump outside the setup applied slight suction through the small holes of the perforated MEA to keep the retina in place, which permits access to the cells from the photoreceptor-side for intracellular recordings (see Figure S1A). In 9 experiments, the eyes were hemisected and the retinas were placed ganglion cell-side-down into the bath chamber on a nitrocellulose filter membrane without a MEA. During the experiment, the retina was superfused with oxygenated (95% O_2_ and 5% CO_2_) Ringer’s medium (110 mM NaCl, 2.5 mM KCl, 1 mM CaCl_2_, 1.6 mM MgCl_2_, 22 mM NaHCO_3_, 10 mM D-Glucose monohydrate) at ∼25°C.

To obtain intracellular recordings from bipolar cells, we used sharp glass microelectrodes (BF120-60-10; shaped with a P-97 Brown/Fleming pipette puller, Sutter Instruments), which allowed penetrating the retina with little damage and provided for fairly long recordings (∼0.5-2 hours). Such long recordings were attainable in the salamander retina because of the comparatively large cell bodies. Microelectrodes were tip-filled with 4% Neurobiotin (dissolved in 0.1M Tris buffer) and backfilled with 3 M KCl solution (resistance 236 ± 101 MΩ, mean ± SD). With the help of a 60x objective, the tip of the microelectrode was placed above the outer segments of the photoreceptors over a MEA recording site that showed high spiking activity to maximize chances of observing ganglion cell responses under current injection into the bipolar cell. Inside the bath solution, the pipette offset was nulled and the microelectrode was slowly inserted into the retina (1-μm steps coaxially to the electrode) until a cell was impaled. The depth of the electrode tip was monitored with the remote control of the micromanipulator (keypad SM5, Luigs & Neumann). The membrane potential of the cell was recorded using a MultiClamp 700B amplifier (Molecular Devices, San Jose, CA) and digitized at 20 kHz (Digidata 1440A, Molecular Devices). For all the described data analyses, the recorded voltage traces were further filtered by a running median (window = 80 data points) and downsampled to 1-ms resolution by taking every 20th data point.

#### Cell-type identification

To assess the morphology of the intracellularly recorded cells, Neurobiotin was injected at the end of a recording with current pulses (blocks of positive and negative pulses of 80-200 pA, 0.5 s current and 2 s break) for 3-7 minutes. Afterward, the retina was carefully removed from the array, fixated with 4% formaldehyde and further processed with Alexa Fluor 488 Streptavidin (Thermo Fisher Scientific, MA, USA) and with To-Pro-3 (Thermo Fisher Scientific, MA, USA) for nucleus staining. The Neurobiotin-filled cells were imaged with a confocal microscope, and two-dimensional representations were constructed with maximum projections over manually chosen regions to best represent the dendrites and axon terminals within the inner and outer plexiform layer (horizontal views, x-y planes) and the vertical view across the bipolar cell (representation in x-z or y-z planes). For Figure S4A, we traced cells with the semi-automatic Fiji plugin Simple Neurite Tracer (Longair et al., 2011; Schindelin et al., 2012) and marked the boundaries of the inner plexiform layer by detecting the nuclei of the ganglion cell layer and of the inner nuclear layer (INL) with the help of To-Pro-3 nucleus staining. The traced morphologies were further analyzed with MATLAB, and the axon stratification depth within the inner plexiform layer (IPL) was computed as the distribution of the relative distances along the axon branches to the INL boundary, normalized so that the boundary to the ganglion cell layer is at a distance of unity (Wu et al., 2000).

When the staining and imaging were successful, bipolar cells were identified based on their bipolar shape, with neurites in the outer and inner plexiform layer (see Figure S1B). Photoreceptors, on the other hand, could be well recognized by their outer segments, and amacrine cells showed neurites only in the inner plexiform layer (see Figures S1D and S1F). In some experiments, the staining failed, or we could not remove the entire retina from the perforated MEA. Thus, we developed additional criteria to identify bipolar cells. We distinguished bipolar cells (BC) from photoreceptors (PR) by the recording depth (morphologically identified PR were observed directly when entering into the retina, mainly at a depth < 100 μm, BC at a depth of 141 ± 40 μm, mean ± SD), the receptive field size (morphologically identified PRs showed smaller RFs, 34 ± 17 μm, than BCs, 76 ± 27 μm, mean ± SD), and the characteristic visual response of photoreceptors to contrast steps. Bipolar cells were distinguished from amacrine cells by observing the response polarity of the simultaneously recorded ganglion cells to positive and negative current pulses (50-500 pA, 500 ms duration, 2 s interval, for 2-4 minutes), which were injected into the intracellularly recorded cell. The spikes of the ganglion cells were extracted by a semi-automatic custom-made spike sorting program, based on a Gaussian mixture model and an expectation-maximization algorithm (Pouzat et al., 2002). If the majority of the retinal ganglion cells responded repeatedly to positive current injection, the intracellular recorded cells were identified as bipolar cells (Asari and Meister, 2012, 2014). If, however, the majority of the ganglion cells responded to negative current, the intracellularly recorded cells were identified as amacrine cells (Asari and Meister, 2014; de Vries et al., 2011). In total, we identified 51 cells as bipolar cells and focused our analysis on 48 cells that showed clear preference to either negative (OFF cells) or positive (ON cells) contrast steps. The remaining 3 cells were excluded due to unclear contrast preference.

#### Visual stimulation

Visual stimuli were generated by a custom-made software written in C++ and OpenGL and presented on a monochromatic gamma-corrected white OLED monitor (eMagin, 800x600 pixels, 60 or 75 Hz refresh rate). The image of the OLED screen was combined with the light path of an upright microscope through a beamsplitter and focused through a custom-made optics system and the 4x objective of the microscope onto the photoreceptor layer. The pixel resolution at the photoreceptor layer was 2.5 μm x 2.5 μm, and the mean light intensity was 2.5 mW/m^2^ in the low photopic range.

#### Analysis of responses to spot stimuli

During the experiment, we first estimated the location over which subsequent spot stimuli were presented (online-determined center parameters). To do so, we shifted a contrast-reversing spot, whose diameter could be manually increased or decreased, over the screen until a position and diameter size were found that maximally stimulated the cell (n = 30 cells). Alternatively, we applied online analysis of responses to spatiotemporal white noise to obtain the receptive field parameters (n = 6 cells; see below).

#### Estimation of optimal spot size (“local spot”)

To estimate the spot size that best stimulated a cell’s receptive field center (“local spot”) for further analyses, we presented spots of black and white contrast steps (100% contrast) at 11 (n = 29 cells) or 13 (n = 3 cells) fixed diameters (between 10-1200 μm) in random order, centered over the online-determined location. The spots were presented for 0.5 s on a background of mean light intensity and separated by 1 s of background illumination. Each spot size was presented on average 8 times. For each trial, we subtracted the average membrane potential measured over the 200 ms prior to the spot, and for each spot size, we averaged the baseline-subtracted responses over trials. For 4 cells, we presented the spots without an interval of background illumination between individual spots and only 6 spot diameters (50, 100, 200, 300, 400, 500 μm). These spots reversed contrast (100%) at 1 Hz for 4 s, with different spot sizes separated by background illumination of 4 s, which was used for computing the baseline membrane potential.

We used the peak of the trial-averaged response per spot size of the preferred contrast and fitted a difference-of-Gaussians model as described by Borghuis et al. (2013):

$$V\left(r\right)=k_{\text{center}}\left(1-exp\left[\frac{-r^{2}}{2{\sigma_{\text{center}}}^{2}}\right]\right)-k_{\text{surr}}\left(1-exp\left[\frac{-r^{2}}{2{\sigma_{\text{surr}}}^{2}}\right]\right)\;$$

Here V is the peak membrane potential, r is the spot radius, *k*_center_ and *k*_surr_ are the maximum response amplitudes of the center and surround, and σ_center_ and σ_sur__r_ parameterize the radius of center and surround. For further spot-based analyses (see below), we then defined the local spot as the spot from the set of tested spot sizes with diameter closest to an estimated receptive field width of 3 σ_center_ (average receptive field width: 104 μm ± 46 μm, mean ± SD, n = 33 cells). For 3 cells, we observed no response saturation or suppression for larger spot diameters but a rather continuous increase in response with every increase in spot size. For those 3 cells, the estimated receptive field width from the fit was much larger than for the other cells (> 250 μm). Thus, to study the responses to a local spot stimulus, we chose for those 3 cells the closest spot size to the diameter computed with spatiotemporal white noise (see below), which was between 100-160 μm.

#### Hyperpolarization index

We analyzed the contrast representation with the local spot determined as described above (Figure 1) as well as with a large spot (500 μm diameter; Figure 8F). For both local and large spots, we computed a hyperpolarization index (HPi) by comparing the peak membrane potential during preferred contrast (depolarization), *V*_dep_, and the minimum membrane potential during non-preferred contrast (hyperpolarization), *V*_hyp_:

$$HPi=\frac{V_{\text{dep}}+V_{\text{hyp}}}{abs\left(V_{\text{dep}}\right)+abs\left(V_{\text{hyp}}\right)},$$

where abs(.) stands for taking the absolute value. The index takes values close to zero for cells that showed equal amounts of hyper- and depolarization, values near unity for cells that did not show any hyperpolarization (i.e., cells with rectified contrast signaling), and occasionally negative values for cells that showed stronger hyperpolarization than depolarization.

#### Sustained-transient index

We computed the sustained-transient index (STi) from the responses to the local spot of preferred contrast (described above). The STi was defined as the ratio of the steady-state response (average membrane potential over the last 50 ms of the spot presentation, *V*_steady state_) and the peak response (*V*_peak_):

$$STi=\frac{V_{\text{steady state}}}{V_{\text{peak}}}$$

Cells with sustained responses (steady state≈peak) have an index near unity, whereas cells with transient responses (steady state≈0), have an index near zero. Note that the 4 cells recorded with contrast-reversing spots and no intermediate background illumination were excluded here (as well as for the latency calculation) because the response onset to the preferred contrast was confounded by the offset response to the non-preferred contrast.

#### Latency

We characterized the latency of the cells from responses to the local spot of preferred contrast (same as for the STi), using two measures: the response onset and time-to-peak. The response onset (Franke et al., 2017) was defined as the time of the first data point after spot onset that exceeded 3 standard deviations of the baseline (measured during the last 200 ms of the preceding background illumination). The time-to-peak was defined as the time from stimulus onset to the time of maximum response (Krieger et al., 2017).

#### Center-surround index

We computed the center-surround index from the spot stimuli described above by comparing the peak response over all spot sizes (up to including 500 μm, *V*_all spots_) with the peak response for a large spot of 500 μm (*V*_large spot_):

$$center-surround\;index=1-\frac{V_{large\;spot}}{V_{all\;spots}}$$

The center-surround index takes values close to zero if the response to the large spot was similar to the maximum response over all spots and values larger than zero if the response was reduced for the large spot.

#### Spatial nonlinearity index

To test for spatially nonlinear stimulus integration, we presented uniform and spatially structured spots (“patterned spots”) at the online-determined center parameters (see above). The patterned spots were obtained by dividing the uniform spot into two halves (“split spot”; n = 33 cells), four quarters (n = 33 cells), or into a checkerboard layout with squares of 25 μm (n = 33 cells) or 10 μm (n = 22 cells), with opposite contrast (±100%) in adjacent stimulus subfields (see Figure 7). The uniform spot and the patterned spots were periodically reversed at 1 Hz for 4 s, followed by 4 s at background illumination. For each voltage trace, the baseline, determined as the mean voltage over the last 200 ms of the preceding background illumination, was subtracted.

To analyze the responses, we computed the average response for one stimulus cycle of 1 s duration (leaving out the first cycle to reduce stimulus onset artifacts), subtracted the mean, and performed a Fourier analysis (MATLAB function *fft*) to obtain a power spectrum. If cells integrated the stimulus over space nonlinearly, both reversals of the spatial patterns could activate the cell, leading to frequency doubling (power at 2 Hz) in the response (Hochstein and Shapley, 1976b). For some cells, we further observed a 4-Hz component in the response to the patterned spots (see Figure S2B). We therefore computed a spatial nonlinearity index (SNi) for each spatial pattern from the combined power in relevant higher harmonics (2 Hz and 4 Hz) of the spatially structured spot, normalized by the power at 1 Hz of the uniform contrast-reversing spot:

$$SNi=\frac{P_{patterned\;spot\;}\left(2\;Hz\right)+P_{\;patterned\;spot\;}\left(4\;Hz\right)}{P_{uniform\;spot\;}\left(1\;Hz\right)}$$

The normalization term acts as a generic measure of response strength of the cell (Turner and Rieke, 2016). Note that for 11 out of 33 cells, the uniform contrast-reversing spot used for normalization was presented with a fixed set of diameters (50, 100, 200, 300, 400, 500 µm), which did not always (8 out of 11 cells) contain the exact diameter size of the patterned spots. For those cells, we therefore linearly interpolated the membrane potential traces from the two uniform spots closest to the diameter size of the patterned spots and then computed the 1-Hz power to obtain the normalization term. Finally, we chose the maximum SNi over the three spatial patterns recorded for all 33 cells (spot with two halves, four quarters, and 25 μm checkerboard) as a representation of the cell’s integration characteristics (Hochstein and Shapley, 1976b). A resulting SNi value near zero means that the cell did not show substantial frequency doubling for any spatial pattern, indicating linear spatial integration.

To study the integration properties under global stimulation, we presented full-field spatial sine-wave gratings with spatial periods of 80, 160, and 300 μm and 8 equally spaced spatial phases each. The gratings were reversed at 1 Hz for 8 s. For each spatial phase, we computed the average response per cycle (leaving out the first cycle to reduce stimulus onset artifact) and computed the Fourier transform as described above to assess the power at 1 Hz and higher harmonics (2 and 4 Hz). For each spatial period, we computed a spatial nonlinearity index (SNi_grating_) by summing the mean power at 2 Hz and 4 Hz, averaged over all phases, and dividing the sum by the maximum power at 1 Hz over all phases (Hochstein and Shapley, 1976b; Petrusca et al., 2007). Finally, we selected the maximum SNi_grating_ over all spatial periods to quantify nonlinear spatial integration under reversing full-field gratings (Hochstein and Shapley, 1976b).

#### Prediction of responses to the split spot

To predict the response to a single reversal in the split spot experiment, we multiplied the averaged trace of the uniform contrast-reversing spot by 0.5 and summed the parts from the first 500 ms (black) and the second 500 ms (white). The prediction for a full cycle of the reversing split spot was then obtained by concatenating two identical 500-ms predictions for a single reversal. Note that the predicted trace was identical for the different spatial patterns (spot with two halves, four quarters, 25 μm or 10 μm checkerboard), because the total amount of black and white contrast inside the receptive field stayed the same. Note also that we did not need to adjust for response latency, which delays the response to the black or white spot compared to the time of the contrast reversal, because the same delay is expected for the reversing patterned spot. The response latency thus simply results in the same temporal phase shift of the periodical signals of the predicted and measured responses under reversing patterned spots.

For comparison with the predicted trace of a given cell, we selected the response trace of the patterned spot (spot with two halves, four quarters, or 25 μm checkerboard) with the highest spatial nonlinearity index because this indicated the most balanced activation by both reversals. To quantify the similarity between the predicted response trace, $V_{predicted}\left(t\right)$, and the measured response, $V_{patterned\;spot}\left(t\right)$, of the cell, we first shifted the predicted response to have the same mean as the measured response in order to account for drifts in the baseline. We then computed the prediction accuracy as follows:

$$prediction\;accuracy=1-\frac{\sum_{t}{\left(V_{patterned\;spot}\left(t\right)-V_{predicted}\left(t\right)\right)}^{2}}{\sum_{t}{\left(V_{uniform\;spot}\left(t\right)-{\bar{V}}_{uniform\;spot}\right)}^{2}}\;,$$

where $V_{uniform\;spot}\left(t\right)$ is the response trace for reversing uniform spots and ${\bar{V}}_{uniform\;spot}$ the corresponding temporal average. The measure is similar to the coefficient of determination, and the adjusted denominator serves to normalize the deviation between predicted and measured response by the scale of a generic response rather than by the response to the patterned spot itself, which may be near zero for a linear cell. The similarity measure takes values near unity if the predicted and measured responses match and values near zero if they are unrelated.

#### Pharmacology with split-spot stimulation

We pharmacologically blocked GABAergic and glycinergic inhibition by adding strychnine (final concentration 5 μM, S0532-5G, Sigma-Aldrich) and picrotoxin (final concentration 100 μM, P1675-1G, Sigma-Aldrich) to the oxygenated Ringer’s solution. As for the patterned-spot experiment (see above), we presented uniform spots and patterned spots composed of two halves (“split spot”) and four quarters. We presented the spots before drug application (“control”), 10 minutes after drug onset (“inhibition block”), and 10 minutes after returning back to the standard Ringer’s solution (“wash-out”). For these recordings, as well as for the measurements under current injection and varying temporal frequencies described below, we optimized the stimulus placement prior to the actual recording by showing contrast-reversing split spots and carefully searching for a spot position for which the responses to both reversals were approximately equal.

#### Current injection with split-spot stimulation

To determine whether the observed nonlinear integration was driven by excitatory or inhibitory inputs, we injected depolarizing and hyperpolarizing currents (±200, ± 300 and ± 400 pA) while presenting uniform and split spots to assess the effects of altered driving force of cationic and anionic synaptic inputs (Zaghloul et al., 2003). The spots were presented individually for 0.5 s, with 4 s of homogeneous background illumination separating successive spot presentations. Current injection started 1 s before spot presentation, allowing the membrane potential to settle to a new level before visual stimulation, and lasted until 150 ms after spot offset. The currents were applied in sequence (+200 pA, −200 pA, +300 pA, −300 pA, +400 pA, and −400 pA), and each spot pattern was presented 2-3 times per current. Prior to this sequence, responses without current injection were obtained with the same visual stimulus sequence. The baseline membrane potential for each current level was obtained by the average potential during the 200 ms prior to the spot presentations. For some cells, some currents induced unstable voltage fluctuations and the responses to those currents were excluded from further analysis. Furthermore, all response traces were filtered with a Gaussian-weighted moving average (SD = 10 ms, 50 ms window). To correct for the voltage drop across the access resistance of the electrode, we recorded in bridge mode and in addition corrected the baseline membrane potential level by the corresponding value in the I-V curve of the electrode, as measured in the bath after the recording (Ashmore and Copenhagen, 1983; Zaghloul et al., 2003). We then plotted the maximum of the trial-averaged light response against the corrected baseline membrane potential (see Figure 5C) and fitted a straight line to estimate the reversal potential from the line’s intercept with the x axis.

#### Input resistance

To obtain an estimate of the cell’s input resistance, we plotted the injected current (I, ± 50 to ± 500 pA) against the baseline membrane potential level (V) and fitted a straight line, from which we obtained the input resistance as the slope. For cells recorded in bridge mode, we used an I-V curve measured with the electrode in the bath to correct for residual, unbalanced electrode resistance; for other cells, we subtracted the resistance of the electrode to estimate the input resistance. The values were around 183 MΩ ± 147 MΩ (mean ± SD, n = 31 cells). Note, though, that measurements with sharp electrodes, unlike with patch electrodes, can only provide a rough estimate of a cell’s input resistance. Furthermore, the set of applied currents varied in our recordings, and some cells were only recorded with two current values. Nevertheless, the range of obtained input resistances roughly agreed with previously reported input resistances in light-adapted goldfish bipolar cells measured with patch electrodes (range 115-384 MΩ; Protti et al., 2000) and were slightly larger than input resistances previously measured with sharp electrodes in carp and goldfish (range 8-64 MΩ; Kujiraoka et al., 1988; Nawy and Copenhagen, 1990).

#### Varying temporal frequencies of split-spot stimulation

As for the patterned-spot experiment (see above), we presented uniform and split spots at different frequencies (0.25, 0.5, 1.0, 2.0, and 3.75 Hz). For each spot pattern and temporal frequency, we computed the trial-averaged response, from which the amplitude modulation was calculated as the difference between the response maximum and minimum. The amplitude modulation was a more stable measure to track the response over the different temporal frequencies than the spatial integration index obtained from the Fourier transform, as lower temporal frequencies yielded less sinusoid-like responses.

#### Spatiotemporal white noise analysis

##### Receptive field estimation

We visually stimulated the retina with binary spatiotemporal white noise in a checkerboard layout, where each square had a size of 30 μm and was updated randomly to black or white (100% contrast; denoted as stimulus values of ±1 Weber contrast for analysis) at 30 (n = 8 cells), 15 (n = 8 cells), 10 (n = 2 cells), or 7.5 Hz (n = 1 cell). For cells for which we also recorded natural movies, the squares had a size of 22.5 μm and were updated at 25 Hz (n = 11 cells) or 12.5 Hz (n = 2 cells) to fit the spatial and temporal resolution of the natural movies (see natural movie subsection below). Recording duration under spatiotemporal white noise was 23 ± 19 minutes (mean ± SD). To remove slow fluctuations in the responses, the voltage traces were first de-trended with a high-pass filter (Butterworth filter, 0.1 Hz cutoff) and then binned at the temporal resolution of the stimulus by computing the average membrane potential per time bin.

The spatiotemporal receptive field was computed in the following way: For each time bin, the average membrane potential was used as a weight for the preceding stimulus sequence (denoted in Weber contrast for each pixel and frame) over two seconds to compute a response-weighted average of all 2 s stimulus sequences, analogous to the common calculation of the spike-triggered average for spiking neurons (Chichilnisky, 2001). From the obtained response-weighted average, we determined the pixel with the largest absolute value over space and time, selected a window around the pixel of 720 μm to the side, and separated the response-weighted average within this window into the highest-ranked spatial and temporal components by singular-value decomposition (Gauthier et al., 2009).

To extract the receptive field location and size, we fitted a two-dimensional Gaussian function to the spatial component. We used the 1.5-sigma contour of the fitted Gaussian for displaying outlines of receptive fields and approximated the receptive field diameter as the diameter of a circle with the same area as within the 1.5-sigma contour. For some cells, we additionally recorded responses to spatiotemporal binary white noise with 10 μm x 10 μm squares to better separate the small receptive field contours of photoreceptors from bipolar cells. We excluded 3 cells out of 32 cells from the spatiotemporal white noise analysis because of noisy receptive fields. We detected noisy receptive fields by computing the average pixel intensity within the 3-sigma boundary of the Gaussian fit from the frame that contained the maximum pixel intensity and checking whether this signal was smaller than the noise level, determined as 3 standard deviations of the values in the spatiotemporal receptive field in the window 2-4 s before the spike.

#### Output nonlinearity index

To assess the degree of nonlinear contrast representation under white noise stimulation, we analyzed the output function (“nonlinearity”) of the linear-nonlinear (LN) model (see Figure 1). The first stage of the model is the linear spatiotemporal filter, which is obtained from the spatiotemporal receptive field. To avoid noise contributions from pixels outside the receptive field, we reduced the number of elements in the spatiotemporal filter by setting pixel values of the response-weighted average outside the 3-sigma contour of the Gaussian fit to zero and re-separating the spatiotemporal receptive field within this window into the highest-ranked spatial and temporal components by singular-value decomposition. Each component was normalized to unit Euclidean norm. We assumed space-time separability and applied the spatial and temporal component of the spatiotemporal receptive field as separate filters. This yielded good approximations of the full spatiotemporal receptive field and helped avoid overfitting by strongly reducing the number of filter parameters. To obtain the output function, we first applied the spatial filter to each frame of the white noise stimulus by computing the scalar product between the spatial filter and the frame’s pixel contrast values and then convolved the resulting temporal sequence with the temporal filter to obtain the linear prediction of the LN model, also called the generator signal. Finally, the output function was obtained as a histogram by binning the generator signal values into 40 bins with equal numbers of data points and averaging the generator signal as well as the corresponding membrane potential values for each bin.

To quantify the degree of nonlinearity in the output function, we computed an output nonlinearity index (ONi) by fitting straight lines separately to the right half of the output function (positive generator signal values) and to the left half (negative generator signal values) and comparing the corresponding slope values *S*_pos_ and *S*_neg_:

$$ONi=\frac{S_{pos}-S_{neg}}{abs\left(S_{pos}\right)+abs\left(S_{neg}\right)}$$

The index takes values close to zero when the two slopes were identical, indicating a linear representation of contrast, whereas values near unity correspond to nonlinear thresholding (i.e., rectification) of negative values of the generator signal. Negative values indicate a weaker response to positive generator signals, which can occur for saturating responses (see Figure 8D).

#### Assessing LN model performance

For 26 cells, we used a non-repeating binary white noise sequence, where 200 segments of 300 stimulus frames duration were randomly chosen as test data. For each test data segment, the LN model (spatial filter, temporal filter, and output function) was obtained from the remaining data (training data). For each stimulus frame of the test segment, the membrane potential was predicted by filtering the preceding stimulus sequence and applying linear inter- and extrapolation of the output function to extract the corresponding membrane potential value. The measured and predicted responses were compared by computing the explained variance (R^2^) as the square of the Pearson correlation coefficient R, and performance was reported as the average R^2^ over all test data segments. For the response traces in Figure 3B, the test segment is shown for which R^2^ was closest to the average R^2^.

For 6 cells, we used a non-repeating binary white noise sequence that was regularly interrupted (every 1200 frames) with an identical sequence of 300 frames (test segment, 10 trials on average). The LN model was obtained from the non-repeated training sequence, and we predicted the response to the test segment. The prediction performance was computed as R^2^ between the predicted and average measured response trace.

#### Temporal filter latency

The time-to-peak of the temporal filter was approximated by fitting a second-order polynomial to 3 data points (at stimulus bin resolution) around the maximum (for ON cells) or minimum (for OFF cells) of the filter and selecting the extremum of the fit (Khani and Gollisch, 2017).

#### Biphasic index

Temporal filters were often biphasic with a second peak at longer latency and opposite sign as compared to the first peak. To quantify this, we calculated a biphasic index as the ratio between the absolute values of the second and first peak of the temporal filter (Khani and Gollisch, 2017; Zaghloul et al., 2007), where the first peak was defined as the maximum for ON cells and minimum for OFF cells and the second peak was the extremum of opposite sign. The index takes values close to zero when the second peak was close to zero (monophasic filter shape) and values of unity when the second peak had the same size as the first peak.

#### Full-field white noise analysis

For 11 cells, we recorded responses to full-field white noise, which homogeneously activates the entire receptive field in a global fashion. Light intensity values were randomly drawn from a Gaussian distribution with a standard deviation of 30% around the same mean light intensity as for the other stimuli and updated at 30 Hz (n = 9 cells) or 25 Hz (n = 2 cells). The stimulus was composed of a non-repeated intensity sequence (training set), which was regularly interrupted (every 900 frames) by the same, repeated sequences of 300 frames (test set, presented on average 13 times). For analysis, light intensity values were converted to Weber contrast (range of −1 to +1). The linear filter and output function of the LN model were obtained from the training set and computed in the same way as for the spatiotemporal white noise, except that the stimulus sequence was one-dimensional and the filter therefore only had a temporal component. The linear filter was normalized to unit Euclidean norm. In Figure 8G, we computed the output nonlinearity index from the output function of the full-field white noise in the same way as described above for spatiotemporal white noise. To assess the performance of the LN model, the response to the test set was predicted by first convolving the test stimulus sequence with the filter and then linearly inter- and extrapolating the output function to obtain the corresponding membrane potential. The performance of the LN model was again quantified by the explained variance (R^2^) between the predicted and the average measured response trace.

#### Natural movie analysis

The natural movies were chosen from the “CatCam” database, where diverse outdoor scenes (e.g., woods, grass) had been recorded with a camera mounted on the head of a cat (Betsch et al., 2004) at 25 Hz and a resolution of 320x240 pixels. The movies had been used previously to study responses in ganglion cells, visual cortex, and lateral geniculate nucleus (Katz et al., 2016; Kayser et al., 2003; Mante et al., 2008). We chose five different movies of 20-40 s duration. For the projection on the retina, movie pixels spanned 3x3 pixels of the display projector to match the resolution of the spatiotemporal white noise, and movies were then cropped to the 800x600 pixels of the display. The light intensity was scaled so that the mean intensity was the same as for the other visual stimuli, and the standard deviation of pixel intensities was near 45% of the mean intensity. Movies were displayed at 25 Hz (except for two cells where the frame rate was reduced to 12.5 Hz) and repeated 9 times on average.

To predict the responses to the natural movies, we applied the LN model as obtained under binary white noise stimulation. The series of frames of the natural movie, represented by the contrast values relative to the overall mean intensity of the movie (Weber contrast, range of −1 to +1), was filtered with the spatial and temporal filters, and the filter output was passed through the corresponding nonlinearity, using inter- and extrapolation, as described above. The model performance was measured by the R^2^ between the averaged movie response and the prediction. From a total of 13 cells, we excluded 3 cells with noisy receptive fields (see spatiotemporal white noise section). Additionally, we excluded 1 cell and the responses to 2 movies from 2 cells, where the baseline membrane potential under the natural movie (average over the last 10 s) had increased by more than 20 mV compared to the starting voltage level under spatiotemporal white noise (average over the first 10 s). For some cells, we observed a drift in the overall response amplitude between the natural movie and the spatiotemporal white noise. For better visual comparison of the temporal profile of response and prediction, we therefore corrected for those offsets by normalizing the displayed predicted traces in Figure 4C to have the same mean and standard deviation as the response to the natural movie.

To quantify the nonlinearity of contrast representation under natural movies, we computed an output nonlinearity directly for the natural movies in the same way as for the white noise data, but using the temporal and spatial filters obtained from white noise (Heitman et al., 2016). Thus, we related the filter output when using the movie as a stimulus to the corresponding measured membrane potential via a histogram (40 bins). The degree of nonlinearity was then quantified by computing the ONi for this output function as explained above. Note that this nonlinearity obtained directly from the natural movie was not used for the response prediction via the LN model.

#### Oscillation frequency

To analyze oscillations that were observed in some recordings, we computed the oscillation frequency from responses to a full-field step in light intensity, where illumination changed alternatingly from background to white (+100% contrast) or to black (−100% contrast), each for 1 s (Figure S4D). For each trial, we subtracted the average membrane potential measured over the 200 ms prior to the light step and computed the average response trace over trials. We applied an analysis window of 800 ms, ranging from 250 ms after stimulus onset to 50 ms after the end of the contrast step and used the mean-subtracted average response to the preferred contrast. The oscillation frequency was determined as the frequency with maximum power, as assessed by Fourier analysis of the response trace in the analysis window.

### Quantification and statistical analysis

Statistical tests for significance were performed with a two-sided Wilcoxon rank sum test (ranksum function in MATLAB) when samples were independent (Figures 8D) and with a two-sided Wilcoxon signed rank test (signrank function in MATLAB) for paired samples (Figure 7B; Figure S2D). Correlation coefficients between two variables were computed as the Pearson correlation coefficient (corrcoef function in MATLAB), whose significance was assessed by a t test. Statistical significance was defined by a p value < 0.05. The statistical details (correlation coefficient, p value, sample size n) are provided in the figures, figure legends, or the text of the Results section. The specific meaning of the sample size n and the definition of center and dispersion measures are clarified when used. The inclusion and exclusion criteria for recorded cells are described in the following subsections of the STAR Methods: Cell-type identification, Sustained-transient index, Spatiotemporal white noise analysis, and Natural movie analysis.

## Supplemental information

Document S1. Figures S1–S4

Document S2. Article plus supplemental information
