# Supplementary material for: Nonlinear spatial integration in retinal bipolar cells shapes the encoding of artificial and natural stimuli
Source: Neuron. 2021 May 19;109(10):1692–1706.e8. doi: 10.1016/j.neuron.2021.03.015 (PMC8153253; doi:10.1016/j.neuron.2021.03.015)
Supplement: Document S1. Figures S1–S4 [file mmc1.pdf]

Neuron, Volume *109*

Supplemental information

Nonlinear spatial integration in retinal bipolar cells shapes the encoding of artificial and natural stimuli

Helene Marianne Schreyer and Tim Gollisch

Figure S1

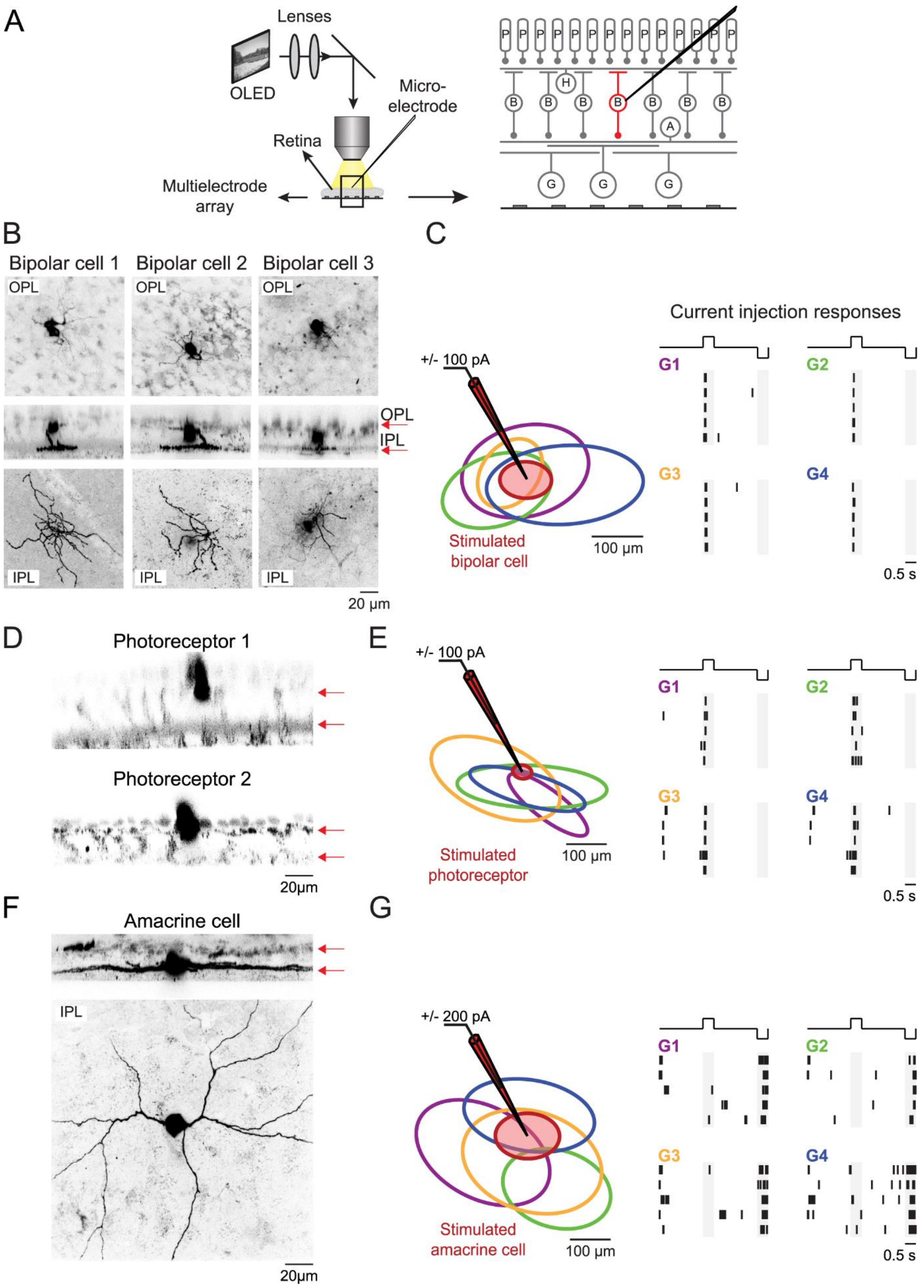
A
Lenses
OLED
Micro-electrode
Retina
Multielectrode array
P
H
B
A
G
B
Bipolar cell 1
Bipolar cell 2
Bipolar cell 3
OPL
IPL
20 µm
C
Current injection responses
+/- 100 pA
Stimulated bipolar cell
100 µm
G1
G2
G3
G4
0.5 s
D
Photoreceptor 1
Photoreceptor 2
20µm
E
+/- 100 pA
Stimulated photoreceptor
100 µm
0.5 s
F
Amacrine cell
IPL
20µm
G
+/- 200 pA
Stimulated amacrine cell
100 µm
0.5 s

Figure S1. **Bipolar cell recordings in the whole-mount retina, Related to Figure 1**

A. Schematic view of the experimental setup. The sharp microelectrodes were inserted from the photoreceptor (P) layer to the bipolar cell (B) somata. Ganglion cells (G) faced a perforated multielectrode array.

B. Sample morphologies of three recorded bipolar cells. The rows show horizontal sections through the outer plexiform layer (OPL; top) and through the inner plexiform layer (IPL; bottom) as well as vertical sections (center; red arrows mark the OPL and IPL location). The images are maximum projections over manually selected regions.

C. Ganglion cell responses to current injection into a bipolar cell. Left: Receptive field outlines of a recorded OFF bipolar cell (red) and four simultaneously recorded ganglion cells. Right: Spike responses of the same four ganglion cells (colors matching to receptive field outlines) to current injection of positive (+100 pA, first gray-shaded region) and negative current (-100 pA, second gray-shaded region) into the bipolar cell (only 5 trials are shown). All four ganglion cells were OFF cells.

D. Two stained photoreceptors (vertical-section view as in B).

E. Receptive field outlines and responses of four retinal ganglion cells (color-coded) to current injection into Photoreceptor 1 (receptive field shown in red). All four ganglion cells were OFF cells.

F. Stained amacrine cell (vertical- and horizontal-section view in the inner plexiform layer, as in B).

G. Receptive field outlines and responses of four ganglion cells to current injection into the amacrine cell. Both the amacrine and ganglion cells were OFF cells, and the ganglion cells responded to negative current injection.

Figure S2

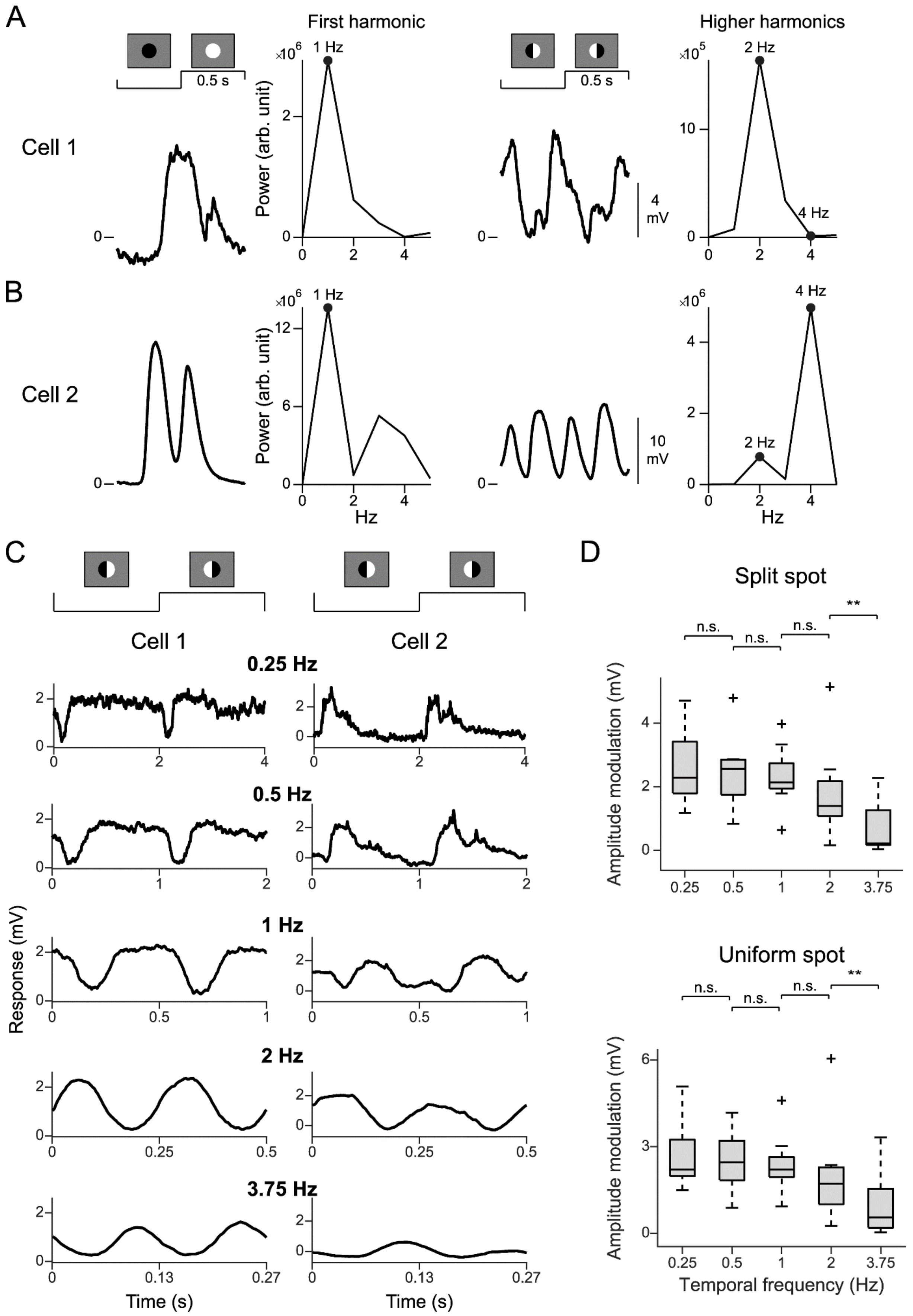

A
First harmonic
Higher harmonics
0.5 s
Cell 1
Power (arb. unit)
4 mV
1 Hz
2 Hz
4 Hz
B
Cell 2
10 mV
Hz
C
Cell 1
Cell 2
0.25 Hz
0.5 Hz
1 Hz
2 Hz
3.75 Hz
Response (mV)
Time (s)
D
Split spot
Uniform spot
n.s.
**
Amplitude modulation (mV)
Temporal frequency (Hz)

Figure S2. **Analysis of spatial nonlinearities, Related to Figure 2**

A. Fourier analysis for computing the spatial nonlinearity index (SNi). Left: Trial-averaged response to the uniform contrast-reversing spot and the corresponding power spectrum of the response for one sample cell. The power at the first harmonic, 1 Hz, is marked with a black dot. Right: Trial-averaged response of the same cell to the split spot and the corresponding power spectrum. The black dots mark the power at higher harmonics (2 and 4 Hz).

B. Same as A for another sample cell. Note the four peaks in the response trace to the split spot (right side) for Cell 2 and the corresponding peak in the power spectrum at 4 Hz.

C. Responses of two sample cells to the split spot presented at different temporal frequencies (0.25 Hz, 0.5 Hz, 1 Hz, 2 Hz and 3.75 Hz). The two cells showed similar modulation amplitudes in the responses to the different temporal frequencies, except for the highest frequency.

D. Distribution of the modulation amplitude for the different temporal frequencies for both the split spot and the uniform spot (n=9 cells, **p<0.01, n.s.= not significant). The reduction in the amplitude at 3.75 Hz can be explained through a general lower sensitivity of the salamander retina to higher temporal frequencies, which is also apparent in the responses to the uniform spot.

Figure S3

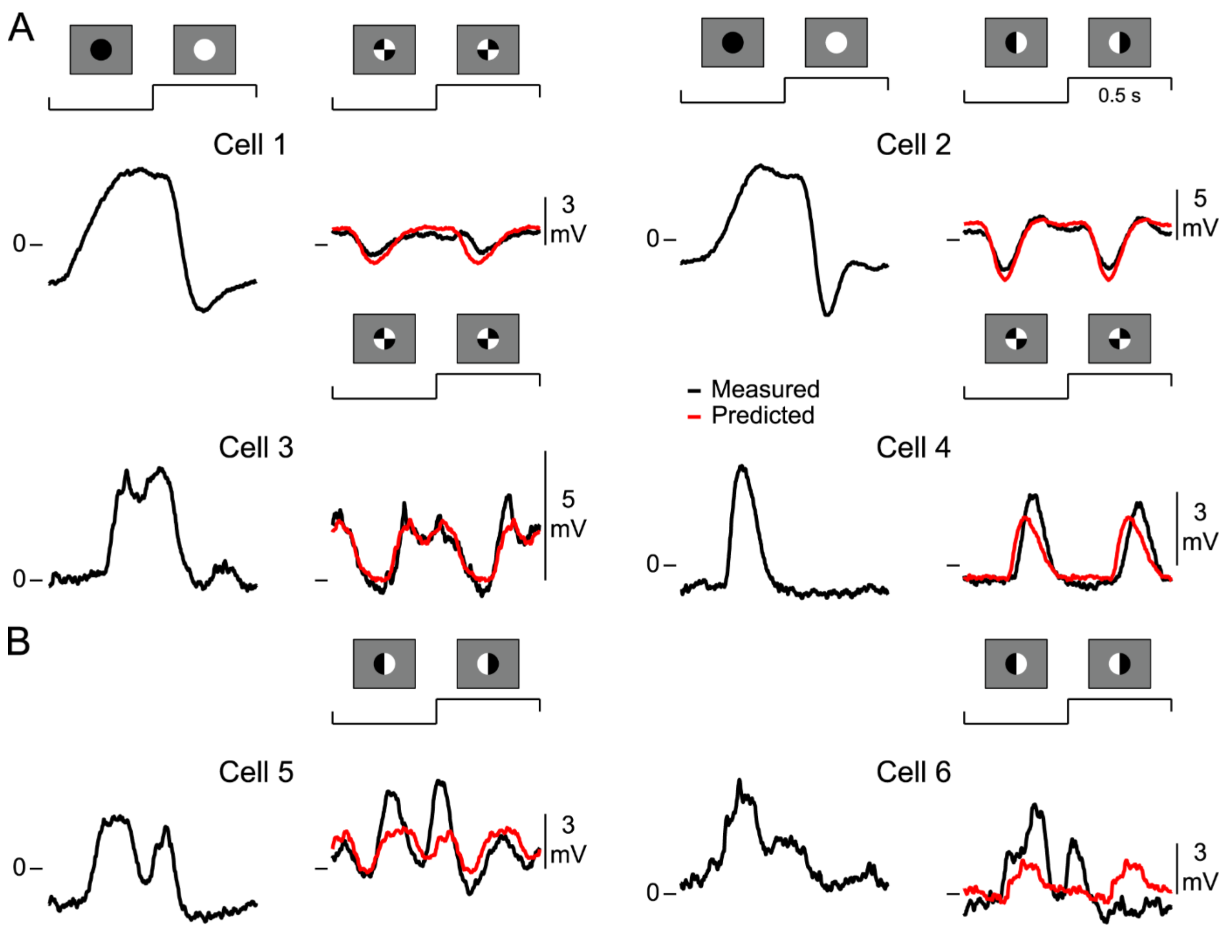

***Figure S3.* Predicting bipolar cell responses to the patterned-spot presentation, Related to Figure 6**

A. Trial-averaged response traces under the uniform contrast-reversing spot and under a patterned spot for four sample cells. The prediction to the patterned spot (cf. Figure 6) is shown in red. The prediction accuracy for all four cells is above 0.7.

B. Same as A for two sample cells where the prediction accuracy is lower (0.59 for Cell 5 and -0.08 for Cell 6). Note that these two cells responded more strongly to the first reversal of the split spot than to the second, which is an indication that the split spot was not well placed to subdivide the receptive field into equally effective subfields. This may explain the lower prediction accuracy for these cells.

Figure S4

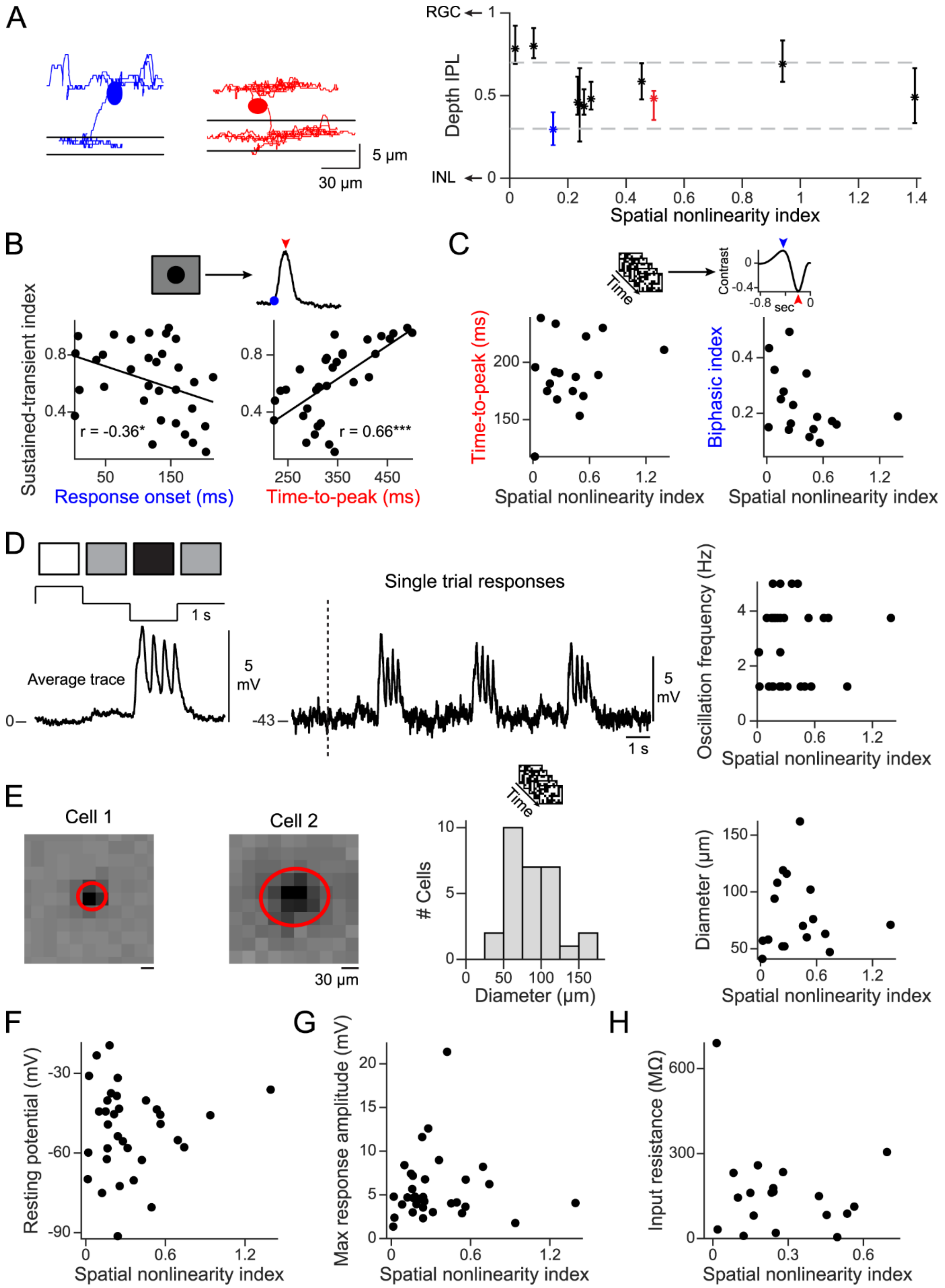
A
RGC
INL
Depth IPL
5 µm
30 µm
Spatial nonlinearity index
B
Sustained-transient index
r = -0.36*
r = 0.66***
Response onset (ms)
Time-to-peak (ms)
C
Time
Contrast
sec
Time-to-peak (ms)
Biphasic index
Spatial nonlinearity index
D
1 s
Average trace
5 mV
Single trial responses
-43
Oscillation frequency (Hz)
Spatial nonlinearity index
E
Cell 1
Cell 2
30 µm
Cells
Diameter (µm)
Diameter (µm)
Spatial nonlinearity index
F
Resting potential (mV)
G
Max response amplitude (mV)
H
Input resistance (MΩ)
Spatial nonlinearity index

Figure S4. **Relation between nonlinear processing and standard response properties in bipolar cells, Related to Figure 8**

A. Left: Morphology of two reconstructed bipolar cells. Black lines mark the boundaries of the inner plexiform layer (IPL). Right: Stratification depth within the IPL. Data points mark the mean stratification level of axonal arborization of individual bipolar cells, the error bars the 25th and 75th percentile (n=11 cells). Unity corresponds to the ganglion cell layer and zero to the inner nuclear layer. The data for the two cells depicted on the left are marked by corresponding colors.

B. Left: Scatter plots of the sustained-transient index (STi) versus response onset (left; r=-0.36, p=0.04, n=32 cells) and versus time-to-peak (right; r=0.66, p=3×10^{-5}, n=32 cells), all computed from responses to the local spot of preferred contrast (cf. Figure 1A). Each black dot marks one bipolar cell, and black lines mark the linear regression (*p<0.05, ***p<0.001).

C. Same as B for the properties of the temporal filter versus the spatial nonlinearity index. No correlation was observed between the spatial nonlinearity index (SNi) and the time-to-peak of the filter (r=0.26, p=0.3, n=17 cells; left) or between the SNi and the biphasic index (r=-0.39, p=0.13, n=17 cells; right).

D. Left: Trial-averaged response trace to full-field contrast light steps that change from gray to white or black in 1-s steps. Note the oscillatory response of the sample cell to the preferred black contrast (~4 Hz). Middle: Single-trial responses of the same cell. The black dashed line marks the onset of positive contrast (“white”). Right: Scatter plot of oscillation frequency versus the SNi. No correlation was observed (r=0.03, p=0.86, n=32 cells).

E. Spatial receptive field components measured with spatiotemporal white noise for two sample bipolar cells and the fitted receptive field outlines (red ellipses). Middle: Distribution of receptive field diameters measured with white noise (range: 41-164 μm, n=29 cells). Right: No correlation was observed between the receptive field diameter and the SNi (r=-0.03, p=0.92, n=17 cells).

F. Comparison of the spatial nonlinearity index and the resting membrane potential. Resting membrane potentials were measured during background illumination prior to the patterned-spot presentations. Each data point corresponds to a different bipolar cell. There was no significant correlation (r=0.04, p=0.8, n=33 cells). Note, though, that measurements of resting membrane potentials (like measurements of input resistance below) are limited in their accuracy in recordings with sharp microelectrodes, owing to effects of potential artificial leak currents.

G. Same as F for the maximum response amplitude under homogenous spots of preferred contrast. No significant correlation was observed (r=0.01, p=0.96, n=33 cells).

H. Same as F for the input resistance and the spatial nonlinearity index. No significant correlation was observed (r=-0.21, p=0.4, n=19 cells).
